# Dissection of genotype × environment interactions for mucilage and seed yield in Plantago species: Application of AMMI and GGE biplot analyses

**DOI:** 10.1371/journal.pone.0196095

**Published:** 2018-05-01

**Authors:** Zolfaghar Shahriari, Bahram Heidari, Ali Dadkhodaie

**Affiliations:** Department of Crop Production and Plant Breeding, School of Agriculture, Shiraz University, Shiraz, Iran; University of Western Sydney, AUSTRALIA

## Abstract

Genotype × environment interaction (GEI) is an important aspect of both plant breeding and the successful introduction of new cultivars. In the present study, additive main effects and multiplicative interactions (AMMI) and genotype (G) main effects and genotype (G) × environment (E) interaction (GGE) biplot analyses were used to identify stable genotypes and to dissect GEI in *Plantago*. In total, 10 managed field trials were considered as environments to analyze GEI in thirty genotypes belonging to eight *Plantago* species. Genotypes were evaluated in a drought stress treatment and in normal irrigation conditions at two locations in Shiraz (Bajgah) for three years (2013-2014- 2015) and Kooshkak (Marvdasht, Fars, Iran) for two years (2014–2015). Three traits, seed yield and mucilage yield and content, were measured at each experimental site and in natural *Plantago* habitats. AMMI2 biplot analyses identified genotypes from several species with higher stability for seed yield and other genotypes with stable mucilage content and yield. *P*. *lanceolata* (G26), *P*. *officinalis* (G10), *P*. *ovata* (G14), *P*. *ampleexcaulis* (G11) and *P*. *major* (G4) had higher stability for seed yield. For mucilage yield, G21, G18 and G20 (*P*. *psyllium*), G1, G2 and G4 (*P*. *major*), G9 and G10 (*P*. *officinalis*) and *P*. *lanceolata* were identified as stable. G13 (*P*. *ovata*), G5 and G6 (*P*. *major*) and G30 (*P*. *lagopus*) had higher stability for mucilage content. No one genotype was found to have high levels of stability for more than one trait but some species had more than one genotype exhibiting stable trait performance. Based on trait variation, GGE biplot analysis identified two representative environments, one for seed yield and one for mucilage yield and content, with good discriminating ability. The identification of stable genotypes and representative environments should assist the breeding of new *Plantago* cultivars.

## Introduction

Medicinal plants with utilization of a very small cultivation area comprise a huge number of plant species [1, 2]. Although only 10% of medicinal species are used commercially, the use of herbal medicines with a diverse array of biological specificities, characteristics and demands is growing [3, 4]. This is due to high costs of developing pharmacological products, increasing human interest in natural and organic products, reported side effects of synthetic materials, perceived environmental pollution by the pharmaceutical industry and the difficulties associated with the discovery of new natural medicines [1, 5–10]. *Plantago* ranks sixth in economical trade of medicinal plants [2, 11] and has been used traditionally for the treatment of liver disease and cancer, stomach troubles, and for inflammation of the bladder and urinary tract [12]. Its mucilage has a mild laxative effect and it is used commercially to cure diarrhea and colds. The mucilage is also used as an excipient for specific drug formulations and has useful carbohydrate polymers for physiologically active drugs [12, 13]. Iran’s geographical location is an ideal environment for the growth and cultivation of *Plantago*. Despite the immense medicinal and export value of *Plantago*, the productivity of its species is constrained by biotic and abiotic stresses which result in heavy losses in seed/husk quality and yield [4, 14]. Moreover, little effort has been devoted to breeding *Plantago* and attempts to expand its genetic variability, cultivation and domestication have had limited success [4, 15–21]. Although consumption of herbal medicines is widespread and increasing, domestication and breeding medicinal plants receives little attention. Global climate change has significant effects on environmental conditions and crop production of all crops. Accordingly, environmental factors affecting plant growth and yield should be understood and managed better for more output [22]. Cultivation under diverse environmental conditions opens up the possibility of using breeding programs to solve problems that are inherent in the production of medicinal plants.

Most plant breeding programs are beset by the challenge of genotype × environment interaction (GEI) [23]. GEI is a key factor in the domestication process and cultivation of plants [1, 9, 24–29]. Approximately 75% of medicinal plant species are collected from the wild and their economic yield is highly affected by environmental conditions [1, 3, 30]. Seed production, secondary metabolite production and their stability in different climates are target traits in which considerable success can be expected simply by selecting vigorous and stable genotypes, a process that establishes a population adapted to the relevant growing conditions [1, 31, 32]. The performance of genotypes is always affected by GEI. Several statistical methods have been proposed for analysis of plant stability with the aim of dissecting GEI and stable trait expression across environments. The simple stability coefficient (P_i_) shows performance of a genotype in multi- environment trials (MET) [33]. This coefficient defines a superior cultivar as one with a performance near the maximum in MET and measures the rank and superiority of a cultivar, as proposed by Lin and Binns [33, 34]. Two frequently used and effective multivariate models for statistical analysis of stability and yield trials are the additive main effects and multiplicative interaction (AMMI) and GGE (genotype (G) main effects and genotype (G) × environment (E) interaction) analysis. These models are used to assess the adaptability and stability of genotypes and to identify mega environments [32, 35–39]. The AMMI model helps to identify genotypes of high productivity adapted to an agronomic zone, with the aim of evaluating environmental [40]. G and GEI are two important sources of variation for evaluating genotypes in MET [31, 32, 39]. AMMI and GGE biplot analyses combine principal component analysis (PCA) and a graphical explanation of GEI. The AMMI model integrates analysis of variance (ANOVA) and PCA as an efficient procedure for an analysis of the stability of genotypes in a MET [39]. It also quantifies the contribution of each genotype and environment to the total GEI variation [40]. GEI can be better understood by analysis of GGE biplots which in turn facilitates the identification of representative environments, detects the ability of test environments to discriminate and identifies stable genotypes in MET [32, 39]. Most studies on medicinal plants have focused on simple analysis of grain yield or secondary metabolites and relatively less effort has been devoted to the advanced analysis of traits in these plants under MET. In addition, few of these analyses have fully dissected GEI or identified representative and discriminating environments to test genotypes [6, 41–44]. Such information provides a better understanding of the GEI being used in breeding programs of medicinal plants, i.e. *Plantago*. The objectives of this study were to (1) dissect GEI for grain yield and mucilage performance of *Plantago* genotypes using AMMI and GGE biplot analyses under various environmental conditions, (2) detect stable genotypes across typical environments and (3) identify representative test environments for future use in breeding programs.

## Materials and methods

### Plant materials

The thirty *Plantago* genotypes used in this study are listed in Table 1. Seeds of each genotype were provided by the Iran Forests, Range and Watershed Management Organization (IFRWMO).

**Table 1:** *Plantago* genotypes, their origin, meteorological data and Köppen-Geiger climate (KGC) classification of the habitat.

Genus	Species	Code	Habitat	Low Temperature (°C)	High Temperature (°C)	Altitude (m)	Latitude (°N)	Longitude(°E)	Mean Temperature (°C)	Annual Rainfall (mm)	KGC*
*Plantago*	*major*	G1	Shazand	-3.3	24.6	1906	33° 56'	49°24'	11.3	312	Dsa
*Plantago*	*major*	G2	Ardebil	-0.2	21.9	1391	38° 14'	48°18'	12.1	381	Csb
*Plantago*	*major*	G3	Sanandaj	-0.4	26.2	1499	32° 25'	47°00'	12.8	492	Csa
*Plantago*	*major*	G4	Shahrood	1.7	25.7	1351	28° 01'	56°21'	14.2	162	Bwk
*Plantago*	*major*	G5	Najafabad	1.9	27.5	1650	32° 49'	51°49'	15	155	Bsk
*Plantago*	*major*	G6	Behshahr	8.5	26.8	16	38° 01'	56°19'	17.3	522	Csa
*Plantago*	*major*	G7	Shiraz	6	28.1	1536	29° 45'	53°00'	16.8	316	Bsk
*Plantago*	*officinalis*	G8	Gorgan	8.2	27.8	133	37° 00'	54°30'	17.8	515	Csa
*Plantago*	*officinalis*	G9	Korramabad	5	29.6	1185	33° 30'	48°30'	16.9	488	Csa
*Plantago*	*officinalis*	G10	Arak	-5.5	26.1	1752	34° 25	49°35'	11.8	316	Dsa
*Plantago*	*amplexicaulis*	G11	Parsabad	0.7	21.9	1391	39° 39'	47°54'	12.1	382	Bsk
*Plantago*	*ovata*	G12	Gonbad	8.3	28.1	164	38° 30	44°54'	17.7	435	Bsk
*Plantago*	*ovata*	G13	Minab	18.4	34.8	42	27° 01	56°54'	27.3	123	Bwh
*Plantago*	*ovata*	G14	Kalale	7.3	28.5	155	37°38'	55°30'	17.4	315	Bsk
*Plantago*	*ovata*	G15	Shiraz	6	28.1	1536	29° 45'	53°00'	16.8	316	Bsk
*Plantago*	*ovata*	G16	Sirmand	11.5	31	928	27° 59'	56°07'	21.7	147	Bwh
*Plantago*	*psyllium*	G17	Dashtestan	14.3	32.6	66	29° 30'	50°45'	24.2	216	Bwh
*Plantago*	*psyllium*	G18	Khoramabad	5	29.6	1185	33° 30'	48°00'	16.9	488	Csa
*Plantago*	*psyllium*	G19	Bandar abbas	18.3	34.3	8	27° 45'	56°00'	27.2	136	Bwh
*Plantago*	*psyllium*	G20	Meshkinshahr	-1.7	21.4	1453	38° 30'	47°50'	9.7	356	Csb
*Plantago*	*psyllium*	G21	Meshkinshahr	-1.7	21.4	1453	38° 30'	47°50'	9.7	356	Csb
*Plantago*	*lanceolata*	G22	Salmas	-2.4	23.3	1378	38° 11'	44°46'	10.5	388	Csa
*Plantago*	*lanceolata*	G23	Estahban	5.7	28.2	1736	29° 07'	54°04'	17	222	Bsk
*Plantago*	*lanceolata*	G24	Markazi	-5.5	26.1	1752	38° 25'	49°35'	11.8	316	Dsa
*Plantago*	*lanceolata*	G25	Semirom	-1.8	24.1	2372	31° 25'	51°34'	11.6	235	Csa
*Plantago*	*lanceolata*	G26	Andimeshk	11.7	36.5	145	32° 31'	48°22'	24.2	358	Bsh
*Plantago*	*coronopus*	G27	Aghghala	8.3	28.4	-14	36° 55'	54° 20'	18.1	432	Bsh
*Plantago*	*coronopus*	G28	Haji abad	11.5	31	928	27°18'	55° 54'	21.7	147	Bwh
*Plantago*	*lagopus*	G29	Dashtestan	14	34.8	66	29° 30'	50° 45'	24.7	211	Bwh
*Plantago*	*lagopus*	G30	Dehloran	9.7	34.4	220	32°41'	47°15'	22.5	280	Bsh

* B: arid, C: warm temperate, D: snow, s: dry summer, w: dry winter, a: hot summer, b: warm summer, k: cold arid, h: hot arid

### Field trials and meteorological data

*Plantago* genotypes were evaluated for mucilage and grain yield under 10 managed field trials. This trait data was compared with trait data collected from *Plantago* genotypes in their natural habitats. The data for the origin of each *Plantago* genotype, its code and the Köppen-Geiger climate (KGC) classification [45] of the experimental sites and natural habitats are presented in Table 1. In the KGC classification system, the letters B, C, D, s, w, a, b, k, and h stand for arid, warm temperate, snow, dry summer, dry winter, hot summer, warm summer, cold arid and hot arid climates, respectively. An environment was defined as the combination of location, year and irrigation regime. Managed field trials were performed in two locations, Shiraz and Marvdasht. In Shiraz, *Plantago* was grown under two irrigation regimes (drought stress and normal irrigation) in three consecutive years (2013-2014-2015) at the Research Farm of School of Agriculture (52° 46′ N, 29° 50′ E). A two-year (2014–2015) experiment under two irrigation regimes was performed at Kooshkak Agricultural Research Station (52° 34′ N, 30° 7′ E), Marvdasht, Iran. The experimental layout in the managed trials in both Shiraz and Kooshkak was a randomized complete block design (RCBD), each with three replications. A normal irrigation regime had normal levels of irrigation throughout growing season while the drought stress treatment was defined as 50% field capacity (FC) at the 2–3 leafs stage of growth. A seed density of 4 kg ha^-1^ was used for sowing in soil at a depth of 4 cm. The 1.2 × 1 m experimental plots consisted of two rows 60 cm apart. Seeds were planted in early April in managed field trials in both Shiraz and Kooshkak. 30 kg N ha^-1^ and 30 kg P_2_O_5_ha^-1^ were added to the soil at sowing. Weeds were controlled manually after seedling emergence and during the growth cycle. Seed yield (g m^-2^), mucilage yield (g m^-2^) and mucilage content (as percentage per 100 g seeds) were measured for each genotype in each trial.

### Mucilage extraction

Mucilage was extracted based on the method of Sharma and Koul [46]. 10ml of HCl (0.1 N) was heated to boiling in a flask and 1 g of *Plantago* seeds was added to it. Heating was resumed and the process of seed husk dissolution was monitored. When all seeds had changed color, the flask was removed from the heat and the solution was filtered through clean muslin cloth, while still hot. The seeds were washed twice with 5 mL of hot water and the solution was filtered. This process helps separation of traces of mucilage. The dissolved mucilage was mixed with 60 mL of 95% ethyl alcohol, stirred and allowed to stand for 5 h. Finally, the supernatant was decanted and the precipitate was dried in an oven at 50° C. This represented the total mucilage content. Mucilage yield (g m^-2^) was also calculated following the method of Sharma and Koul [46].

### Statistical analysis

#### Analysis of AMMI model

The AMMI model for the i^th^ genotype in the j^th^ environment is [35, 47],

$$Y_{ijr}=\mu +g_{i}+e_{j}+b_{r}\left(e_{j}\right)+\sum_{n=1}^{k}\lambda_{k}\alpha_{ik}\gamma_{jk}+\rho_{ij}+\varepsilon_{ij}$$

where, Y_ijr_ is the mucilage or yield of genotype *i* in environment *j* for replicate *r*, *μ* is the grand mean, *g*_*i*_ is the deviation of genotype *i* from the grand mean, *e*_*j*_ is the environment main effect as deviation from *μ*, *λ*_*k*_ is the singular value for the interaction principal component (IPC) axis *k*, *α*_*ik*_ and *γ*_*jk*_ are the genotype and environment IPC scores (i.e. the left and right singular vectors) for axis *k*, *b*_*r*_(*e*_*j*_*)* is the effect of the block *r* within the environment *j*, *r* is the number of blocks, ρ_ij_ is the residual containing all multiplicative terms not included in the model, n is the number of axes or IPC that were retained in the model, and ε_ijr_ is error under independent and identically distribution assumptions,

$$\varepsilon_{ij}\sim \left(N,\frac{\delta^{2}}{r}\right)$$

AMMI Stability Value (ASV) was calculated using the formula developed by Purchase et al. [48]:

$$ASV=\sqrt{{\left[\frac{SS\;IPCA1}{SS\;IPCA2}\left(IPCA1score\right)\right]}^{2}+{\left(IPCA2score\right)}^{2}}$$

where, SSIPCA1 is sum of squares of interaction principal component analysis 1 (IPCA1) and SSIPCA2 is sum of squares of IPCA2.

Sum of the absolute value of the IPC (SIPC) was calculated by a formula developed by Sneller et al. [49],

$$SIPC=\sum_{1}^{n}\left|IPCA_{n}\right|$$

The biplot graph of the AMMI1 (IPCA1 scores vs. additive main effects from genotypes and environments) and AMMI2 (IPCA1 vs. IPCA2) were constructed.

P_i_ is the sum of squares of differences of mean genotype *i* in each environment from the mean of the best genotype in the corresponded environment [33, 34],

$$P_{i}=\frac{[n\;{\left(\bar{X}-\bar{M}\right)}^{2}+\sum_{j+1}^{n}{\left(X_{ij}-{\bar{X}}_{i.}-M_{j}+\bar{M}\right)}^{2}}{2n}$$

where,

$${\bar{X}}_{i.}=\sum_{j=1}^{n}\frac{X_{ij}}{n}\;\text{and}\;\bar{M}=\sum_{j=1}^{n}\frac{M_{j}}{n}$$

${\overset{-}{X}}_{i.}$ is the yield mean of the i ^th^ cultivar in the n environments and M is the mean of the maximum response in the n environments. According to Lin and Binns [33], the first part of the Pi expression quantifies the genetic deviation and the second quantifies GEI. Mean rank of each genotype in all environments was calculated as genotype mean rank.

#### GGE biplot analysis

Singular value decomposition (SVD) of the first two principal components was used to fit the GGE biplot model [50],

$$Y_{ij}=\mu +\beta j+\lambda_{1}\xi_{i1}\eta_{j1}+\lambda_{2}\xi_{i2}\eta_{j2}+\varepsilon_{ij}$$

where, Y_ij_ is the trait mean for genotype i in environment j, μ is the grand mean, βj is the main effect of environment j, μ + β_j_ being the mean yield across all genotypes in environment j, λ_1_ and λ_2_ are the singular values (SV) for the first and second principal components (PC1 and PC2), respectively, ξ_i1_ and ξ_i2_ are eigenvectors of genotype i for PC1 and PC2, respectively, η_1 j_ and η_2 j_ are eigenvectors of environment j for PC1 and PC2, respectively, ε_ij_ is the residual associated with genotype i in environment j. In GGE biplot analysis, scores of PC1 were plotted against PC2 [32].

The square root transformed form of the data for mucilage content(%) was used in statistical analyses. The AMMI and GGE biplot analyses were performed by GENSTAT software V. 12.0 (GENSTAT 2009) [51]. The mean for environments and genotypes compared using the Least Significant Differences (LSD) test in Statistical Analysis System (SASV 9.2).

## Results

### Weather conditions and meteorological data at the trial sites and natural habitats

Meteorological data showed that the *Plantago* genotypes experienced hot temperatures exceeding 30 °C and low rainfall in all experimental trials (Table 2). Two experimental trials at Shiraz received higher annual rainfalls rather than the other 3 trials. Both experimental sites are classified as Bsk in the Köppen-Geiger climate (KGC) classification [45]. Bsk stands for cold arid regions with dry summers. Annual rainfall varied between 214 mm in 2015 at Kooshkak and 373 mm in 2013 at Shiraz. In *Plantago*, rainfall in the natural habitat regions varied between 123 mm for Minab as a natural habitat of *P*. *ovata* and 522 mm for Behshahr as one of habitats of *P*. *major*. The meteorological data shows that *Plantago* species can grow in a wide range of altitudes from below -14 m to above 2000 m.

**Table 2:** Location of the managed field trials and their Köppen-Geiger climate classifications.

Environment	Location	Year	Irrigation regime	Altitude(m)	Latitude (N)	Longitude (E)	Low temperature (°C)	High temperature (°C)	Mean temperature (°C)	Annual rainfall (mm)	Climate classification*
E1	Bajgah (Shiraz)	2013	Normal	1810	29° 50'	52° 46'	-5.8	41.8	17.7	373	Bsk
E2	Bajgah (Shiraz)	2013	Drought	1810	29°50'	52° 46'	-5.8	41.8	17.7	373	Bsk
E3	Bajgah (Shiraz)	2014	Normal	1810	29°50'	52° 46'	-4.4	41.8	18.3	224.8	Bsk
E4	Bajgah (Shiraz)	2014	Drought	1810	29°50'	52° 46'	-4.4	41.8	18.3	224.8	Bsk
E5	Bajgah (Shiraz)	2015	Normal	1810	29°50'	52° 46'	-3.6	40.4	18.8	300	Bsk
E6	Bajgah (Shiraz)	2015	Drought	1810	29°50'	52° 46'	-3.6	40.4	18.8	300	Bsk
E7	Kooshkak (Marvdasht)	2014	Normal	1650	30° 07'	52° 34'	-5	41	17.9	227.5	Bsk
E8	Kooshkak (Marvdasht)	2014	Drought	1650	30° 07'	52° 34'	-5	41	17.9	227.5	Bsk
E9	Kooshkak (Marvdasht)	2015	Normal	1650	30° 07'	52° 34'	-4.8	40.6	18.2	241	Bsk
E10	Kooshkak (Marvdasht)	2015	Drought	1650	30° 07'	52° 34'	-4.8	40.6	18.2	241	Bsk

*Köppen-Geiger climate classification [45] letter: B: arid, s: summer dry, k: cold arid.

### AMMI analysis of variance

The results of AMMI analysis showed that seed yield, mucilage yield and mucilage content were significantly affected by genotype, environment and GEI (Table 3). The AMMI analysis of variance indicated that 53.57% of the total sum of squares (SS) for seed yield was captured by the effect of genotype (G) and 30.25% and 14.72% of the total SS were attributable to the environmental (E) effects and GEI, respectively. For mucilage yield, 86.11% of the total sum of squares is justified by G, 5.17% by E, and 7.22% by GEI. G, E and GEI contributed to 91.54%, 1.63% and 4.96% of the total mucilage content variation, respectively. The IPCA9, IPCA8 and IPCA7 were the last significant IPCAs for seed yield, mucilage yield and mucilage content, respectively (Table 3). AMMI2 (IPCA1 + IPCA2) explained 82.05% of the GEI sum of squares for seed yield, 75.77% for mucilage yield and 53.07% for mucilage content (Table 3).

**Table 3:** AMMI analysis of variance of 30 *Plantago* genotypes for seed yield, mucilage yield and content.

Source	DF*	Seed yield			Mucilage yield			Mucilage content		
		MS	% of total SS	% of total interactions SS	MS	% of total SS	% of total interactions SS	MS	% of total SS	% of total interactions SS
Genotype	29	1707.2**	53.57		114.3**	86.11		15.324**	91.54	
Environment	9	3106.1**	30.25		22.14**	5.17		0.88**	1.63	
Interactions	261	52.1**	14.72		1.06**	7.22		0.092**	4.96	
IPCA1	37	224.6**		61.08	4.45**		59.25	0.238**		36.6
IPCA2	35	81.5**		20.97	1.31**		16.52	0.113**		16.47
IPCA3	33	23.8**		5.78	0.61**		7.24	0.105**		14.4
IPCA4	31	20.2**		4.6	0.59**		6.59	0.089**		11.49
IPCA5	29	15.3**		3.26	0.3**		3.17	0.065**		7.88
IPCA6	27	8.5**		1.68	0.3**		2.88	0.061**		6.85
IPCA7	25	6.8**		1.25	0.21**		1.91	0.028**		2.9
IPCA8	23	5.6**		0.94	0.17*		1.44	0.022^ns^		2.12
IPCA9	21	2.8^ns^		0.43	0.13^ns^		1.01	0.015^ns^		1.29
Residuals	0	0			0			0		
Error	580	2.3			0.1			0.015		

*DF: degree of freedom, MS: mean squares, ns: non- significant,

**: significant at 1% probability level, SS: sum of squares

### Discriminating ability and representativeness of environments

The mean and IPCA1 scores of environments and the top rankings genotypes for each environment are displayed in Table 4. The mean comparison of environments showed that drought stress conditions significantly decreased seed yield compared with a normal irrigation regime and with natural habitat conditions. The mean seed yield (36.23 g m^-2^) and mucilage yield (3.96 g m^-2^) were higher in natural habitats of *Plantago* than means in each of the trial environments (Table 4). Natural habitats yielded higher mucilage content (10.8%) than E6, E7 and E9. However, mean mucilage yield was not significantly decreased under drought stress conditions (E2, E4, E6, E8 and E10). Similarly, mucilage content was not significantly different in drought versus irrigated conditions, although slight increases in content were observed in some drought environments (i.e. E4, E8 and E10) compared with normal irrigation trials (i.e. E3, E7 and E9), respectively, and slight decreases observed in others -E2 and E4, compared with E1 and E3, respectively. In the present study, IPCA1 scores showed that E1, E7 and E6 were main contributors to the stability of genotypes for mucilage yield, seed yield and mucilage content, respectively. Equal zero IPCA1 score indicates highest contribution to genotypes stability, but small contribution to the GE interaction [52, 53].

**Table 4:** Environmental means, IPCA1 score, first four AMMI genotype selections based on seed yield, mucilage yield and content.

Trait	Environment	Mean	IPCA1**	1^st^	2^nd^	3^rd^	4^th^
Seed Yield (g m^-2^)	E4	23.03	3.168	G13	G16	G12	G15
	E6	21.97	2.24	G15	G13	G16	G12
	E10	21.03	2.103	G13	G16	G12	G15
	E8	20.2	1.973	G12	G15	G13	G16
	E2	21.49	1.576	G16	G13	G15	G14
	E7	30.91	-1.19	G13	G15	G12	G14
	E9	30.29	-1.666	G15	G13	G16	G12
	E3	34.56	-2.458	G13	G16	G12	G15
	E1	33.37	-2.753	G15	G14	G13	G16
	E5	32.78	-2.993	G15	G13	G12	G16
	Habitat	36.23					
	*LSD (1)%	5.5577					
Mucilage Yield (g m^-2^)	E10	2.662	1.03	G16	G12	G15	G13
	E6	2.498	0.8525	G15	G13	G12	G16
	E4	2.764	0.84	G13	G15	G16	G12
	E8	2.554	0.7525	G15	G13	G16	G14
	E2	2.652	0.7237	G16	G15	G14	G13
	E1	3.405	-0.5525	G15	G16	G14	G13
	E9	3.525	-0.7167	G15	G12	G13	G16
	E7	3.714	-0.8015	G15	G13	G16	G14
	E3	3.588	-0.8431	G16	G13	G14	G15
	E5	3.502	-1.2848	G15	G16	G13	G14
	Habitat	3.964					
	*LSD (1)%	1.3319					
Mucilage content (%)	E10	11.36	1.0358	G15	G12	G16	G14
	E9	10.06	0.3198	G14	G12	G15	G13
	E8	11.84	0.141	G14	G15	G16	G13
	E6	10.4	0.0012	G24	G15	G23	G13
	E7	9.92	-0.057	G15	G16	G20	G13
	E2	11	-0.1576	G14	G15	G16	G26
	E5	11.12	-0.1847	G16	G15	G14	G13
	E1	11.32	-0.2001	G15	G25	G16	G14
	E3	10.89	-0.3671	G16	G14	G25	G15
	E4	11.25	-0.5312	G15	G25	G22	G13
	Habitat	10.8					
	*LSD (1)%	2.988					

*Differences between environments means for each trait that are equal to or less than the LSD (1%) is not significant.

** Lower IPCA1 score indicates higher environmental stability.

**Fig 1:**
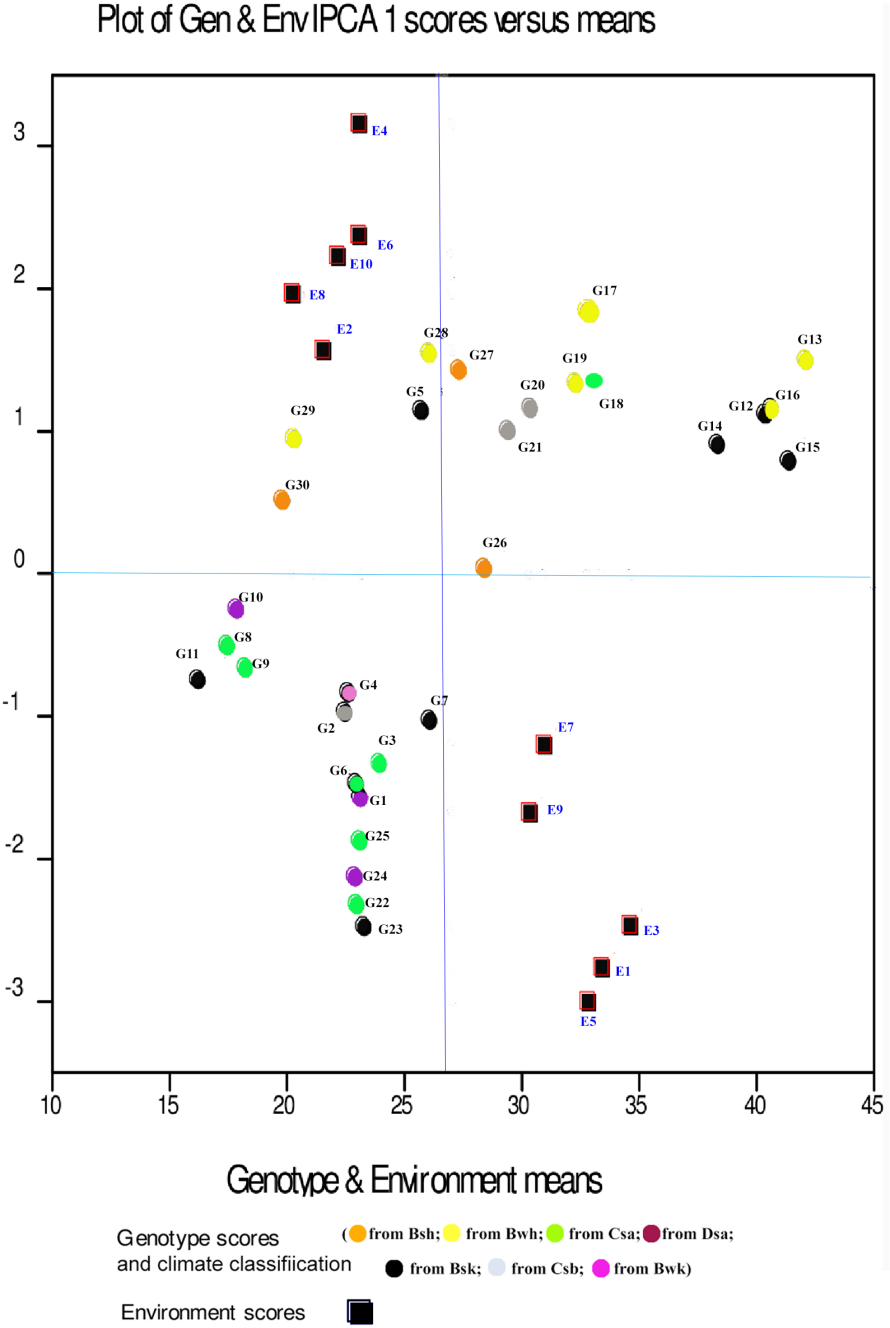
AMMI1 biplot for seed yield (g m^-2^) in 30 *Plantago* genotypes (*P*. *major*, G1- G7; *P*. *officinalis*, G8-G10; *P*. *amplexicaulis*, G11; *P*. *ovata*, G12-G16; *P*. *psyllium*, G17-G21; *P*. *lanceolata*, G22-G26; *P*. *coronopus*, G27- G28; *P*. *lagopus*, G29- G30) and 10 environments (E). Köppen-Geiger climate classification [45] letter: B: arid, s: summer dry, k: cold arid.

**Fig 2:**
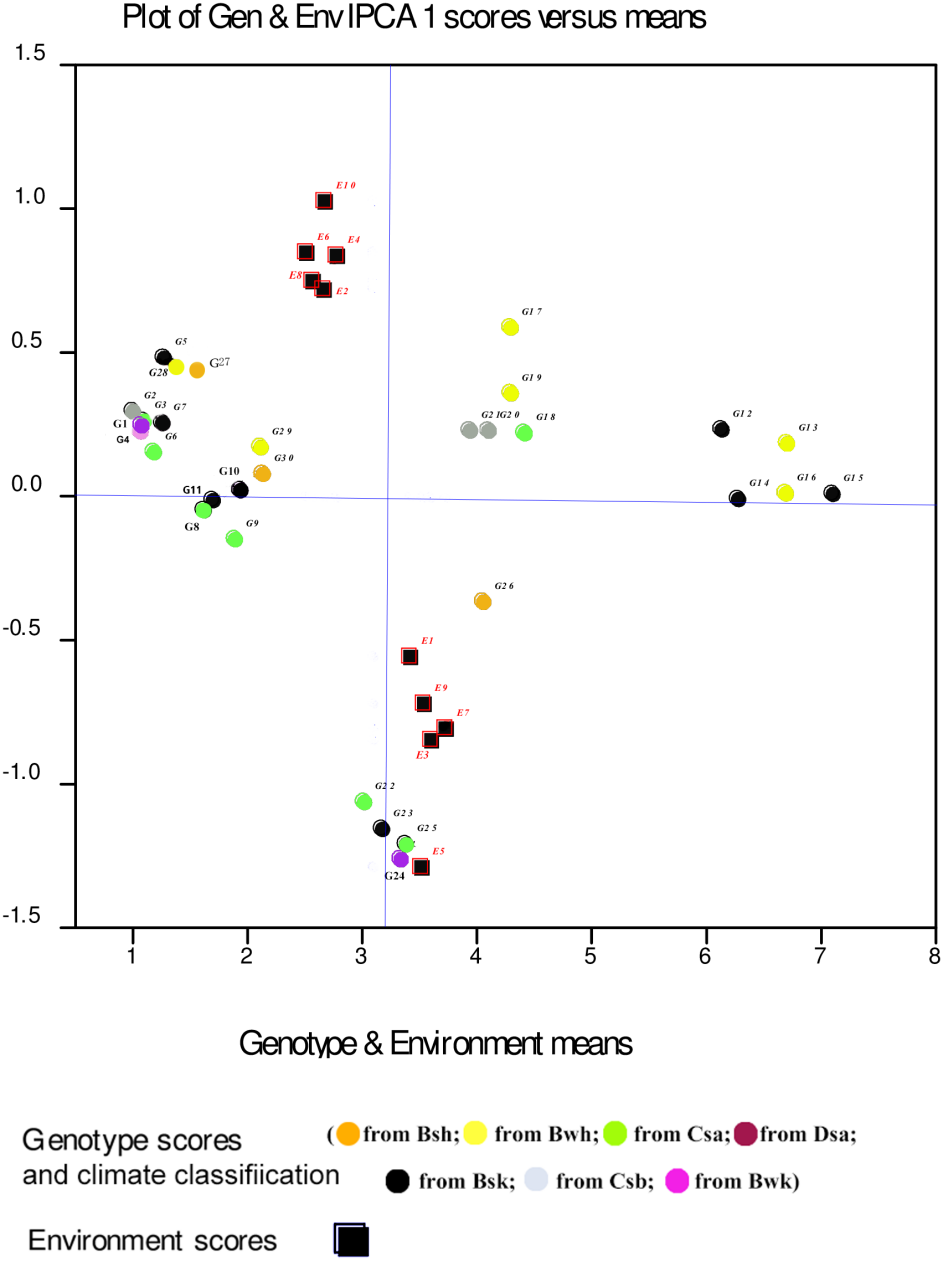
AMMI1 biplot for mucilage yield (g m^-2^) of 30 *Plantago* genotypes (*P*. *major*, G1- G7; *P*. *officinalis*, G8-G10; *P*. *amplexicaulis*, G11; *P*. *ovata*, G12-G16; *P*. *psyllium*, G17-G21; *P*. *lanceolata*, G22-G26; *P*. *coronopus*, G27- G28; *P*. *lagopus*, G29- G30) and 10 environments (E). Köppen-Geiger climate classification [45] letter: B: arid, s: summer dry, k: cold arid.

**Fig 3:**
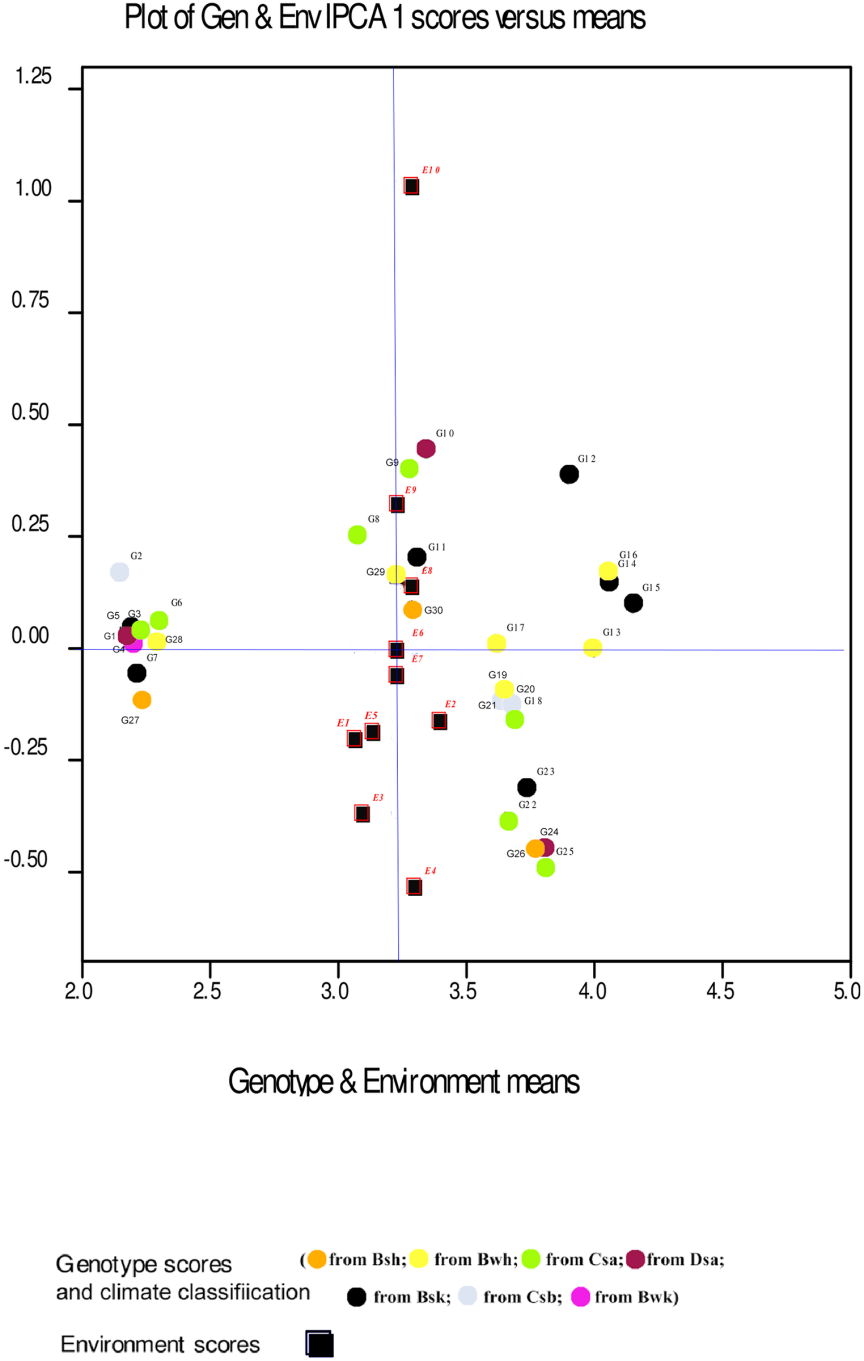
AMMI1 biplot for mucilage content (% of 100 g seed) of 30 *Plantago* genotypes *P*. *major*, G1- G7; *P*. *officinalis*, G8-G10; *P*. *amplexicaulis*, G11; *P*. *ovata*, G12-G16; *P*. *psyllium*, G17-G21; *P*. *lanceolata*, G22-G26; *P*. *coronopus*, G27- G28; *P*. *lagopus*, G29- G30) and 10 environments (E). Köppen-Geiger climate classification [45] letter: B: arid, s: summer dry, k: cold arid.

**Fig 4:**
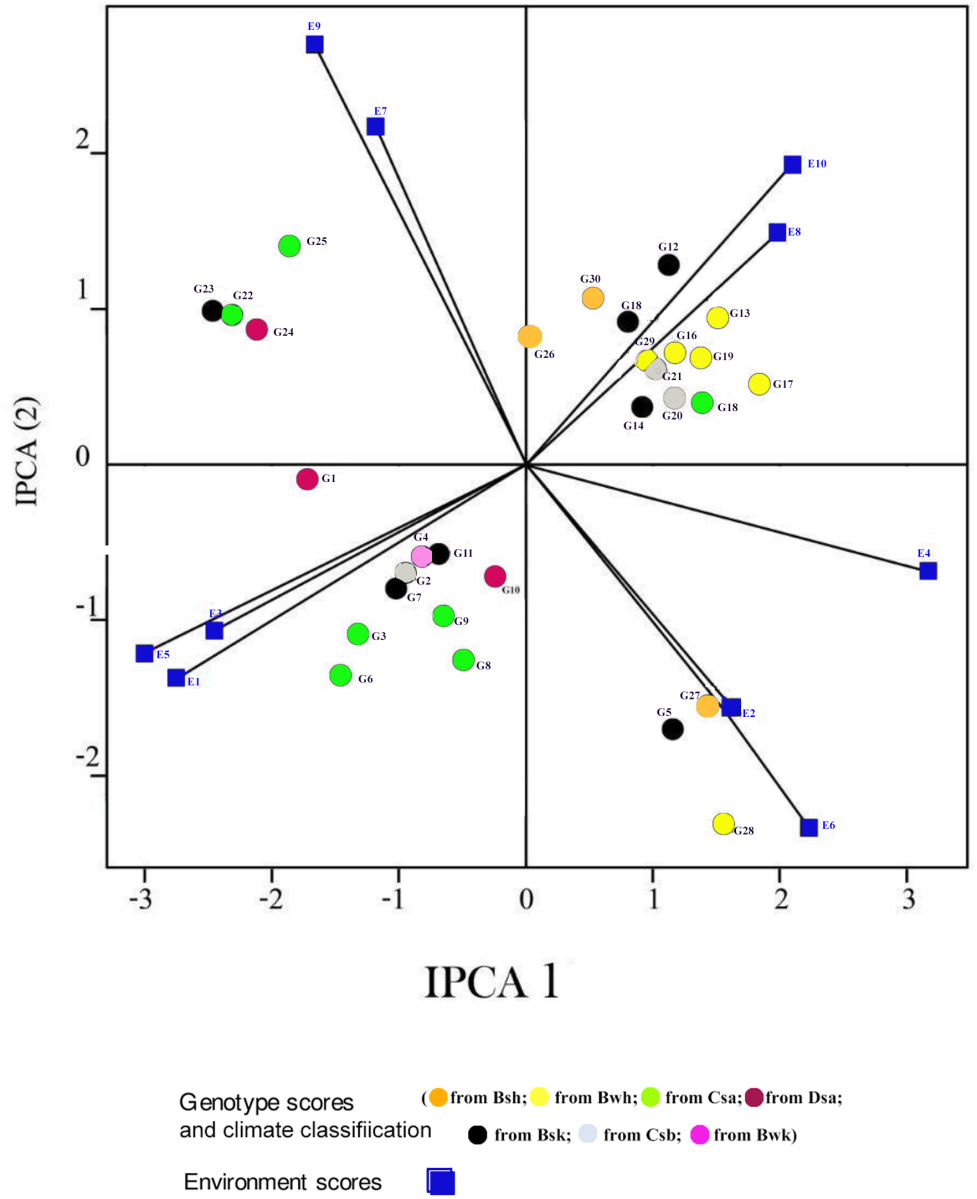
AMMI2 biplot for seed yield (g m^-2^) of 30 *Plantago* genotypes *P*. *major*, G1- G7; *P*. *officinalis*, G8-G10; *P*. *amplexicaulis*, G11; *P*. *ovata*, G12-G16; *P*. *psyllium*, G17-G21; *P*. *lanceolata*, G22-G26; *P*. *coronopus*, G27- G28; *P*. *lagopus*, G29- G30) and 10 environments (E). Köppen-Geiger climate classification [45] letter: B: arid, s: summer dry, k: cold arid.

**Fig 5:**
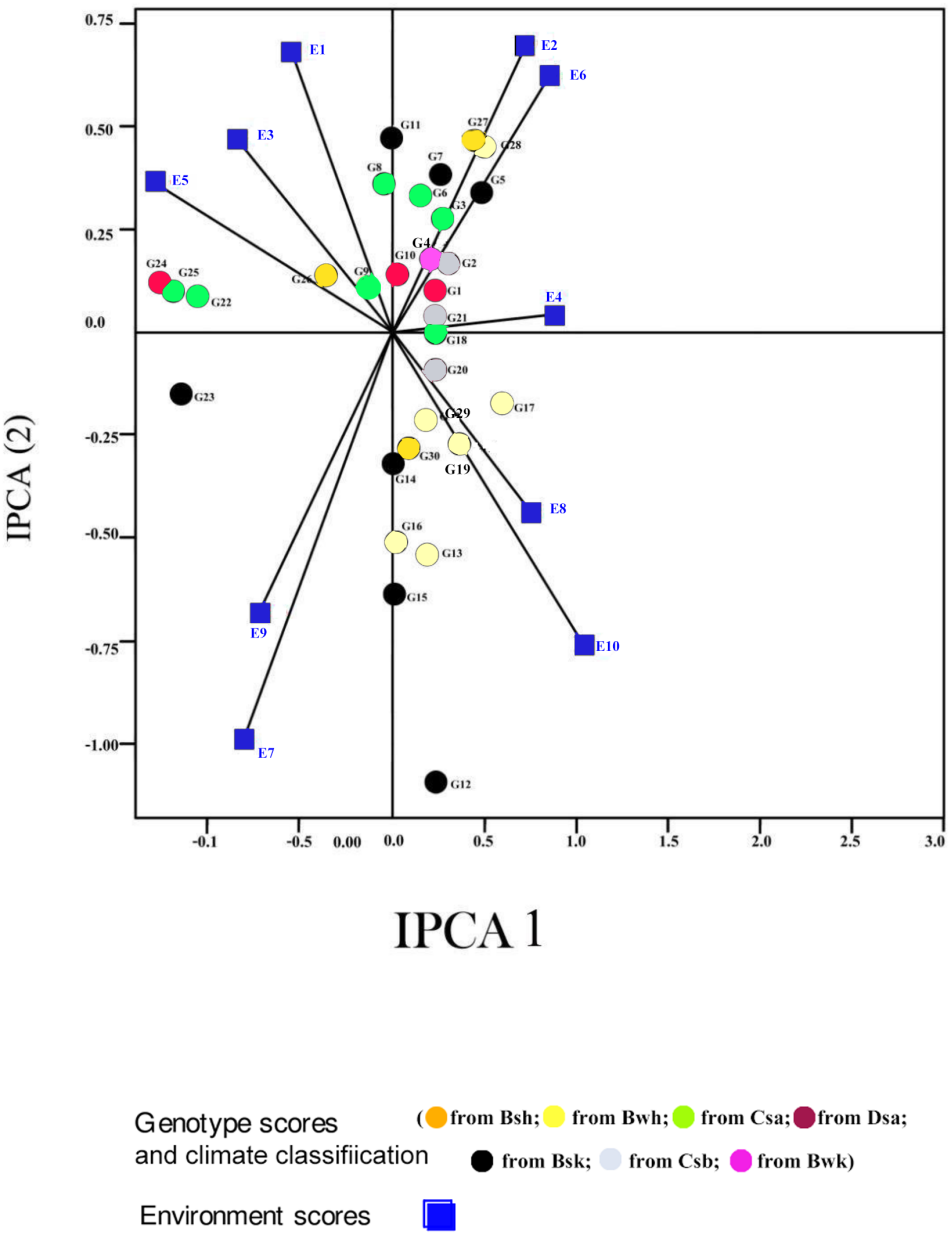
AMMI2 Biplot for mucilage yield (g m^-2^) of 30 *Plantago* genotypes (*P*. *major*, G1- G7; *P*. *officinalis*, G8-G10; *P*. *amplexicaulis*, G11; *P*. *ovata*, G12-G16; *P*. *psyllium*, G17-G21; *P*. *lanceolata*, G22-G26; *P*. *coronopus*, G27- G28; *P*. *lagopus*, G29- G30) and 10 environments (E). Köppen-Geiger climate classification [45] letter: B: arid, s: summer dry, k: cold arid.

**Fig 6:**
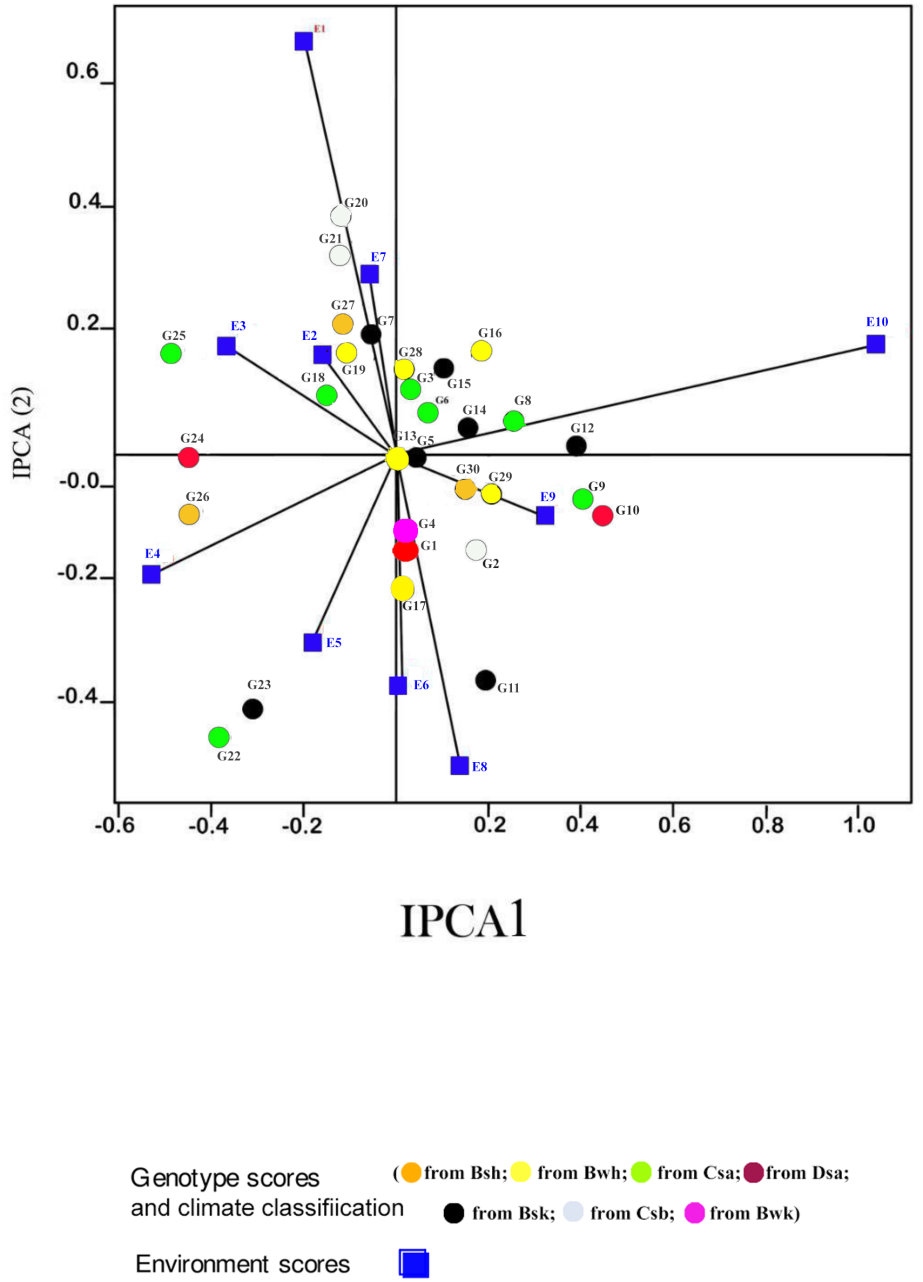
AMMI2 Biplot for mucilage content (per 100 g seeds) of 30 *Plantago* genotypes (*P*. *major*, G1- G7; *P*. *officinalis*, G8-G10; *P*. *amplexicaulis*, G11; *P*. *ovata*, G12-G16; *P*. *psyllium*, G17-G21; *P*. *lanceolata*, G22-G26; *P*. *coronopus*, G27- G28; *P*. *lagopus*, G29- G30) and 10 environments (E). Köppen-Geiger climate classification [45] letter: B: arid, s: summer dry, k: cold arid.

Displacement along the abscissa of AMMI1 biplot graphs indicates main additive effects, whereas displacement along the ordinate shows interaction effects [53]. Environments with IPCA1 scores nearly or equal to zero have small contribution to the interactions and accordingly have large contribution to the stability of genotypes [52, 53]. The AMMI1 biplot graph showed that E7 for seed yield, E1 for mucilage yield and E6 and E7, E2, E8 for mucilage content were the largest contributor to the stability of genotypes (Figs 1, 2 and 3; S1, S2 and S3 Tables). The pattern of dispersion and contribution of environments to genotype stability in the AMMI1 biplot graph for mucilage content was completely different from those for seed and mucilage yield (Figs 1, 2 and 3). The AMMI2 biplot graphs show environment scores for seed yield, mucilage yield and content (Figs 4, 5 and 6). In AMMI2, environments which placed near the origin with low scores for IPCA1 and IPCA2 had small contribution to the GE interaction, but large contribution to the stability of genotypes. AMMI2 showed that all environments were far from the biplot origin for seed yield. It also revealed E2, E7 and E9 as relatively high contributor to the stability of genotypes for mucilage content and E4 and E8 for mucilage yield.

**Fig 7:**
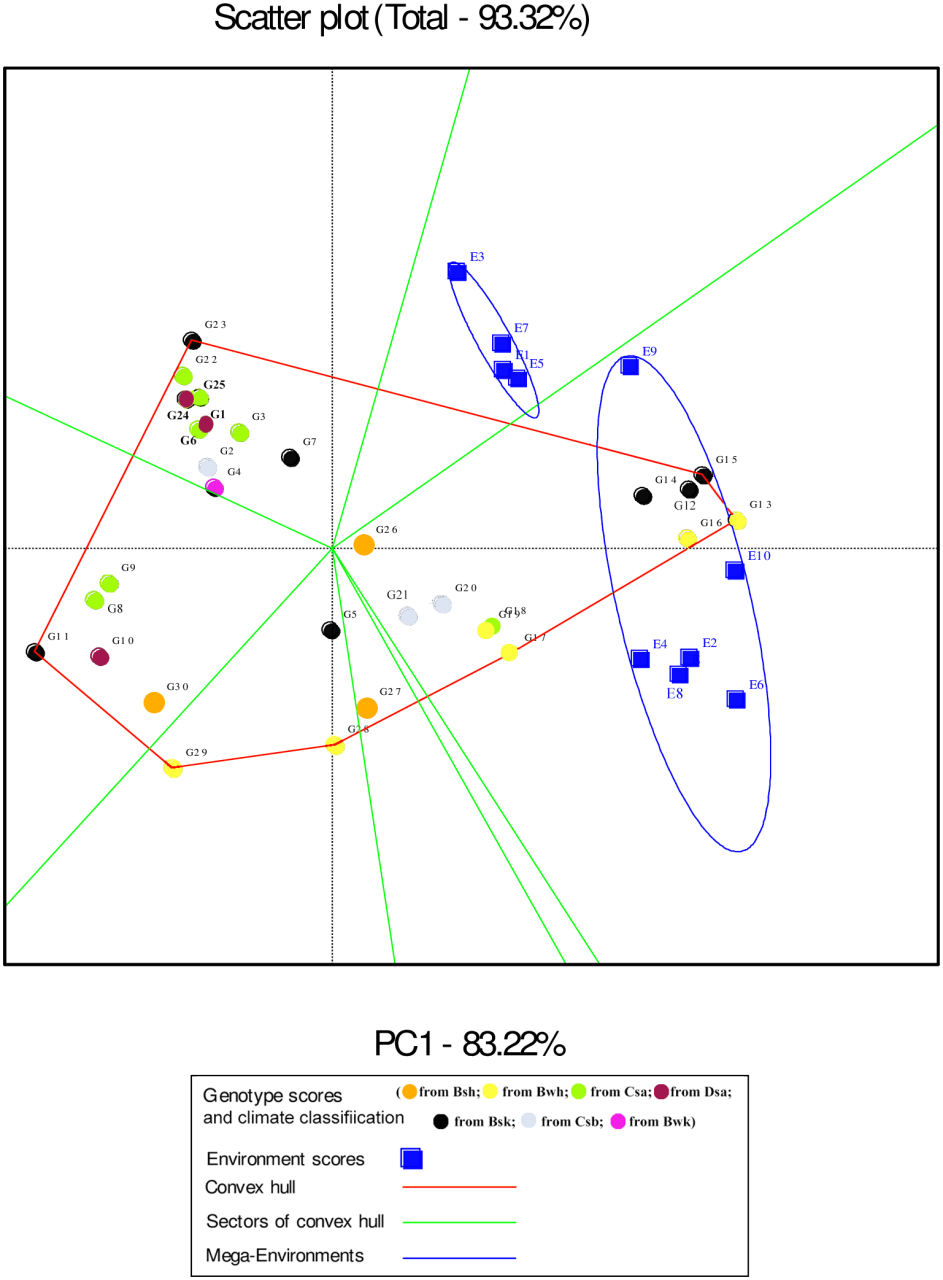
The which-won-where polygon view of the GGE biplot for seed yield of 30 *Plantago* genotypes (*P*. *major*, G1- G7; *P*. *officinalis*, G8-G10; *P*. *amplexicaulis*, G11; *P*. *ovata*, G12-G16; *P*. *psyllium*, G17-G21; *P*. *lanceolata*, G22-G26; *P*. *coronopus*, G27- G28; *P*. *lagopus*, G29- G30) under 10 environments (E) to show which genotype performed best in which environment and meaningful mega environment. The perpendicular of the polygon facilitates the visualize comparison of distance between genotypes and environments. Different mega environments located in different biplot sectors, Köppen-Geiger climate classification [45] letter: B: arid, s: summer dry, k: cold arid.

**Fig 8:**
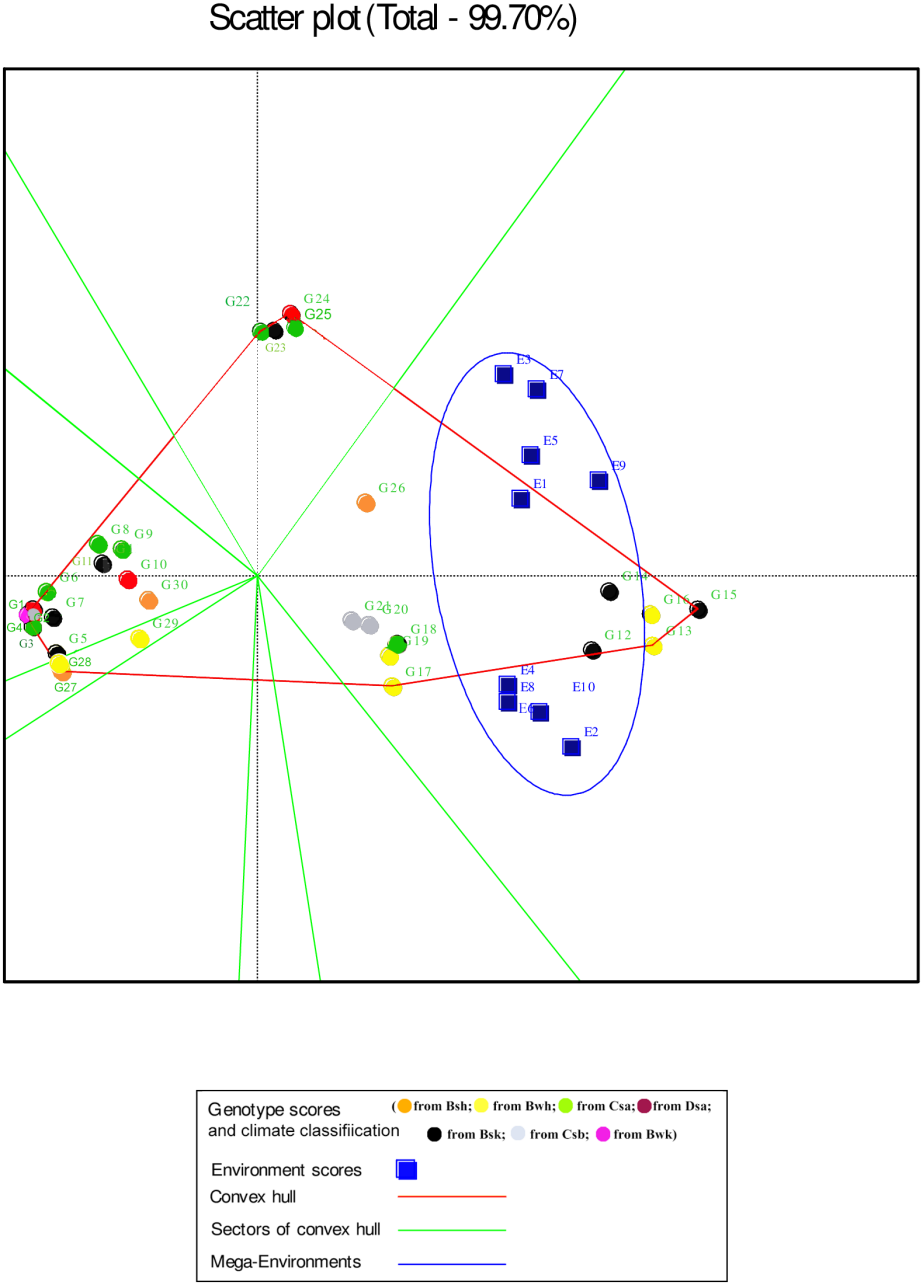
The which-won-where polygon view of the GGE biplot for mucilage yield of 30 *Plantago* genotypes (*P*. *major*, G1- G7; *P*. *officinalis*, G8-G10; *P*. *amplexicaulis*, G11; *P*. *ovata*, G12-G16; *P*. *psyllium*, G17-G21; *P*. *lanceolata*, G22-G26; *P*. *coronopus*, G27- G28; *P*. *lagopus*, G29- G30) under 10 environments (E) to show which genotypes performed best in which environment and meaningful mega environment. The perpendicular of the polygon facilitates the visualize comparison of distance between genotypes and environments. Different mega environments located in different biplot sectors. Köppen-Geiger climate classification [45] letter: B: arid, s: summer dry, k: cold arid.

**Fig 9:**
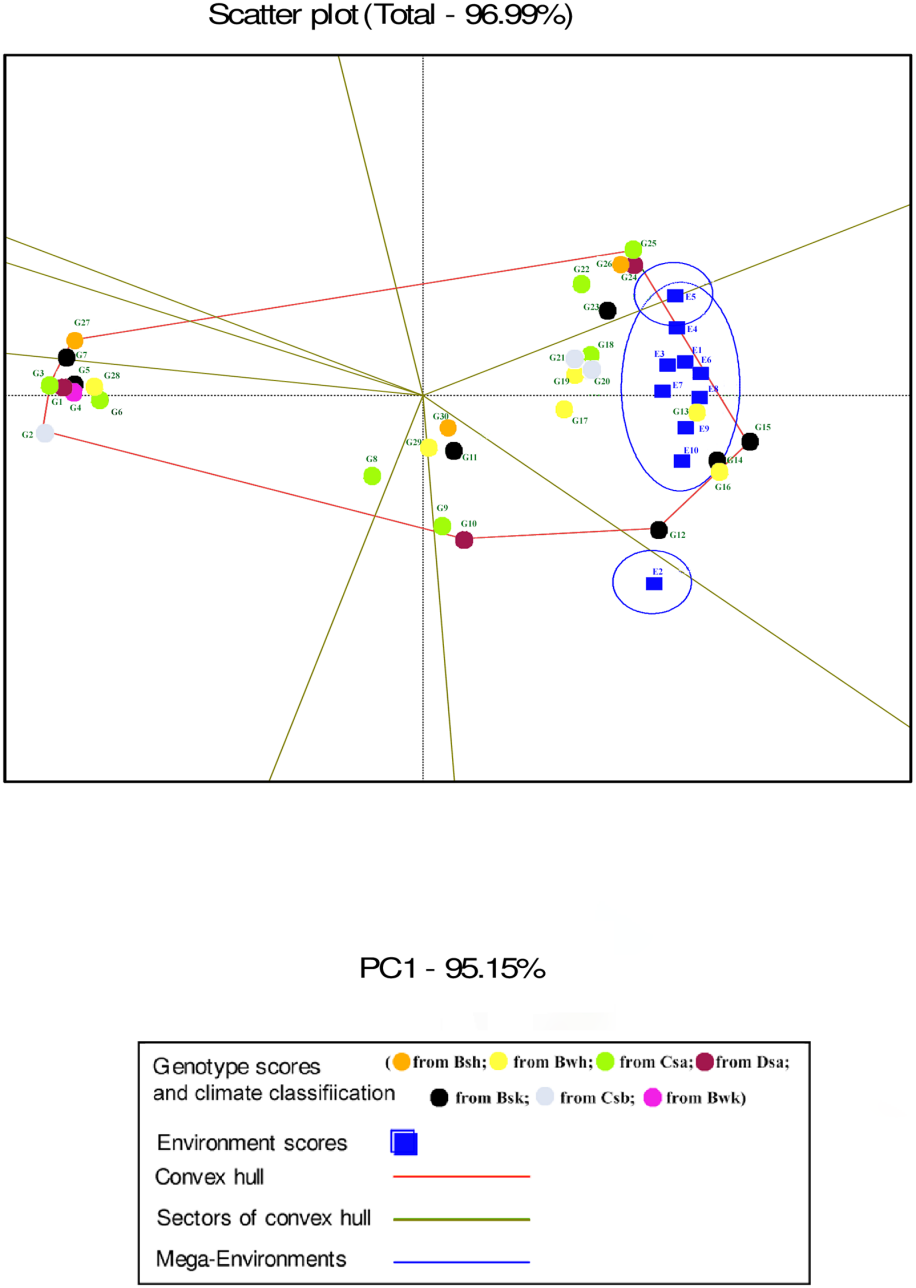
The which-won-where polygon view of the GGE biplot for mucilage content of 30 *Plantago* genotypes (*P*. *major*, G1- G7; *P*. *officinalis*, G8-G10; *P*. *amplexicaulis*, G11; *P*. *ovata*, G12-G16; *P*. *psyllium*, G17-G21; *P*. *lanceolata*, G22-G26; *P*. *coronopus*, G27- G28; *P*. *lagopus*, G29- G30) under 10 environments (E) to show which genotypes performed best in which environment and meaningful mega environment. The perpendicular of the polygon facilitates the visualize comparison of distance between genotypes and environments. Different mega environments located in different biplot sectors. Köppen-Geiger climate classification [45] letter: B: arid, s: summer dry, k: cold arid.

In the GGE biplot analysis, the first two PCs explained 93.32%, 99.7% and 96.99% of the total GEI for seed yield, mucilage yield and mucilage content, respectively (Figs 7, 8 and 9; S4 Table). Environments with low IPCA1 and IPCA2 scores which were placed near the origin in the GGE biplot graph have low discriminating ability for genotypes evaluation and high contribution to the stability of genotypes [32, 39]. As repeatable interactions divide the target environments into mega environments which discriminate genotypes, genotypes should separately be evaluated for each mega environment [32, 39]. In the “which-won-where” GGE biplot graph (Figs 7, 8 and 9), a polygon is drawn by connecting genotypes that are farthest from the biplot origin and all genotypes are surrounded by the polygon. The “which-won-where” GGE biplot graph divided by equality line into sectors in which different mega environments can be detected [31, 32]. In the present study, two mega environments were detected for seed yield. The first mega environment included E1, E3, E5 and E7 whilst the second included E2, E4, E6, E8, E9 and E10 (Fig 7).

In the GGE analysis, genotypes were grouped into a single mega environment for mucilage yield (Fig 8). Distances between the environmental vectors show the dissimilarity of environments in discriminating genotypes [32]. Although environments cannot be divided into meaningful mega- environments as they are scattered in one sector of the which-won-where biplot graph, the distance between environments for mucilage yield was based on drought conditions (Fig 8). E2 was identified as a distinct mega-environment for mucilage content in 2013. In 2013, precipitation and mean temperature was different from 2014 and 2015 in Shiraz which contributed to the separation of E2 from other environments in 2013 (Table 2). E5 and E4 were the second mega-environment, and E1, E3, E6, E7, E8, E9 and E10 were the third for mucilage content (Fig 9).

**Fig 10:**
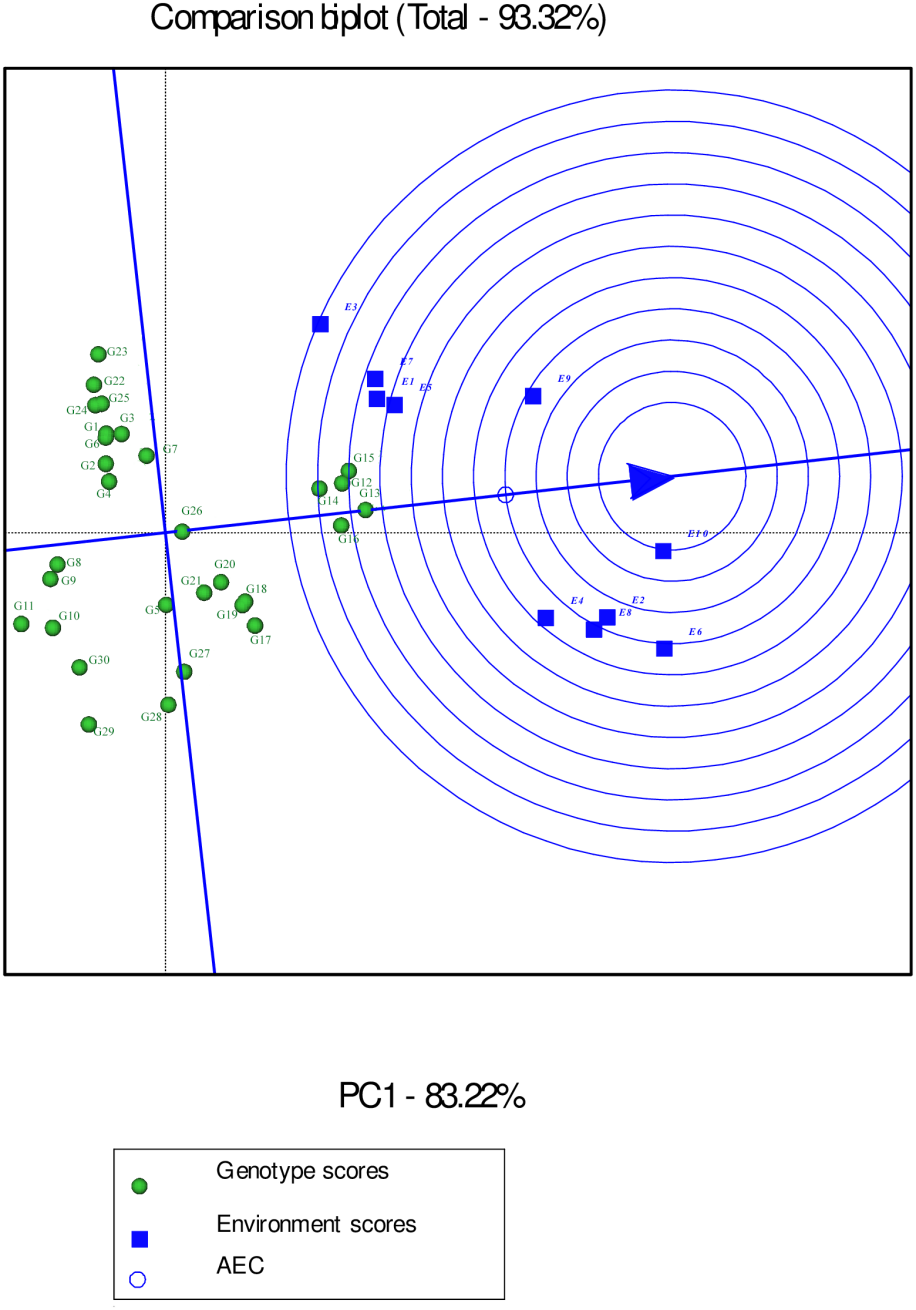
The discrimination and representativeness view of the GGE biplot based on environment-focused scaling for seed yield of 30 *Plantago* genotypes (*P*. *major*, G1- G7; *P*. *officinalis*, G8-G10; *P*. *amplexicaulis*, G11; *P*. *ovata*, G12-G16; *P*. *psyllium*, G17-G21; *P*. *lanceolata*, G22-G26; *P*. *coronopus*, G27- G28; *P*. *lagopus*, G29- G30) under 10 environments (E) to rank test environments relative to an ideal test environments (represented by center of concentric circles).

**Fig 11:**
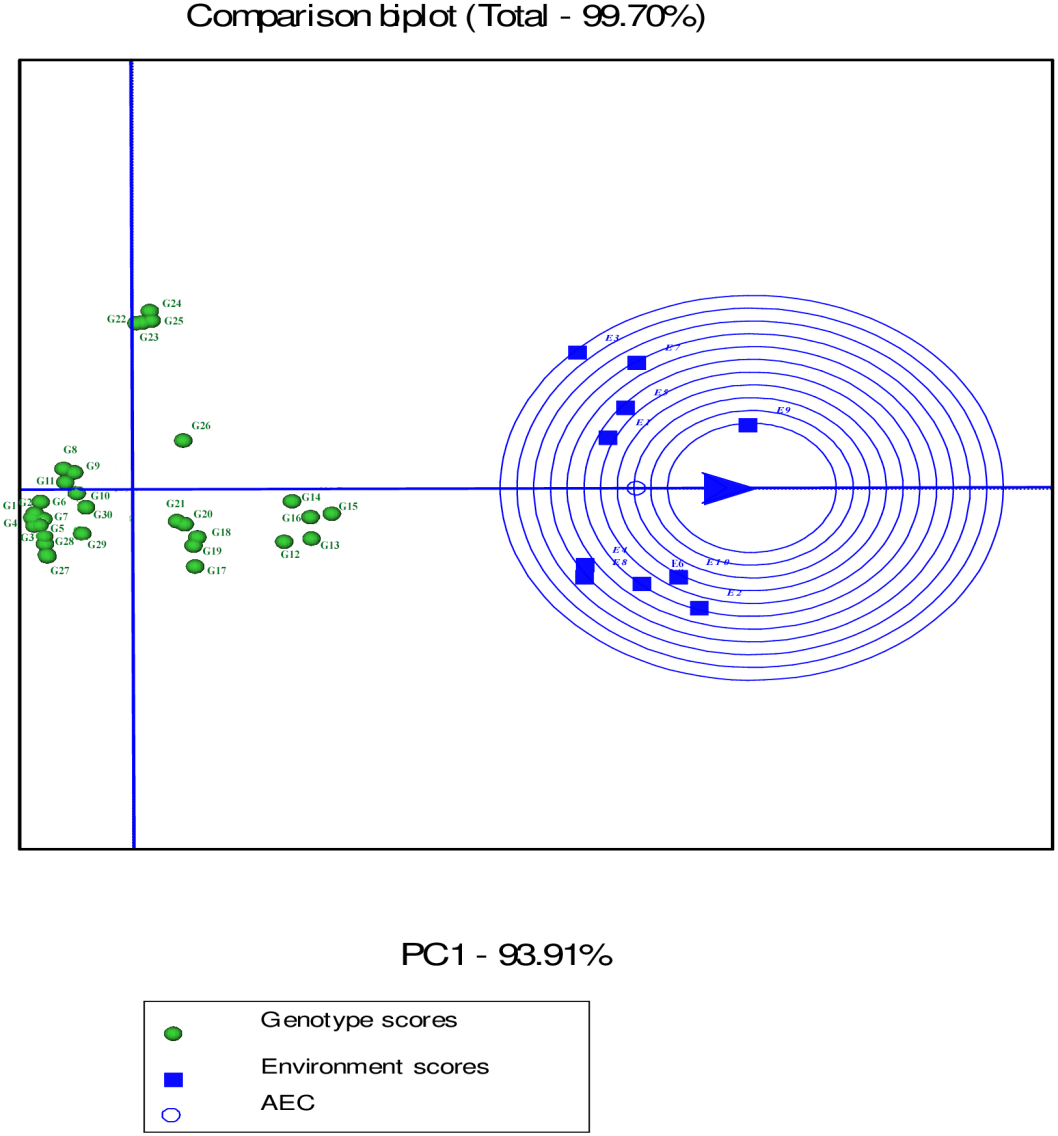
The discrimination and representativeness view of the GGE biplot based on environment-focused scaling for mucilage yield of 30 *Plantago* genotypes (*P*. *major*, G1- G7; *P*. *officinalis*, G8-G10; *P*. *amplexicaulis*, G11; *P*. *ovata*, G12-G16; *P*. *psyllium*, G17-G21; *P*. *lanceolata*, G22-G26; *P*. *coronopus*, G27- G28; *P*. *lagopus*, G29- G30) under 10 environments (E) to rank test environments relative to an ideal test environments (represented by center of concentric circles).

**Fig 12:**
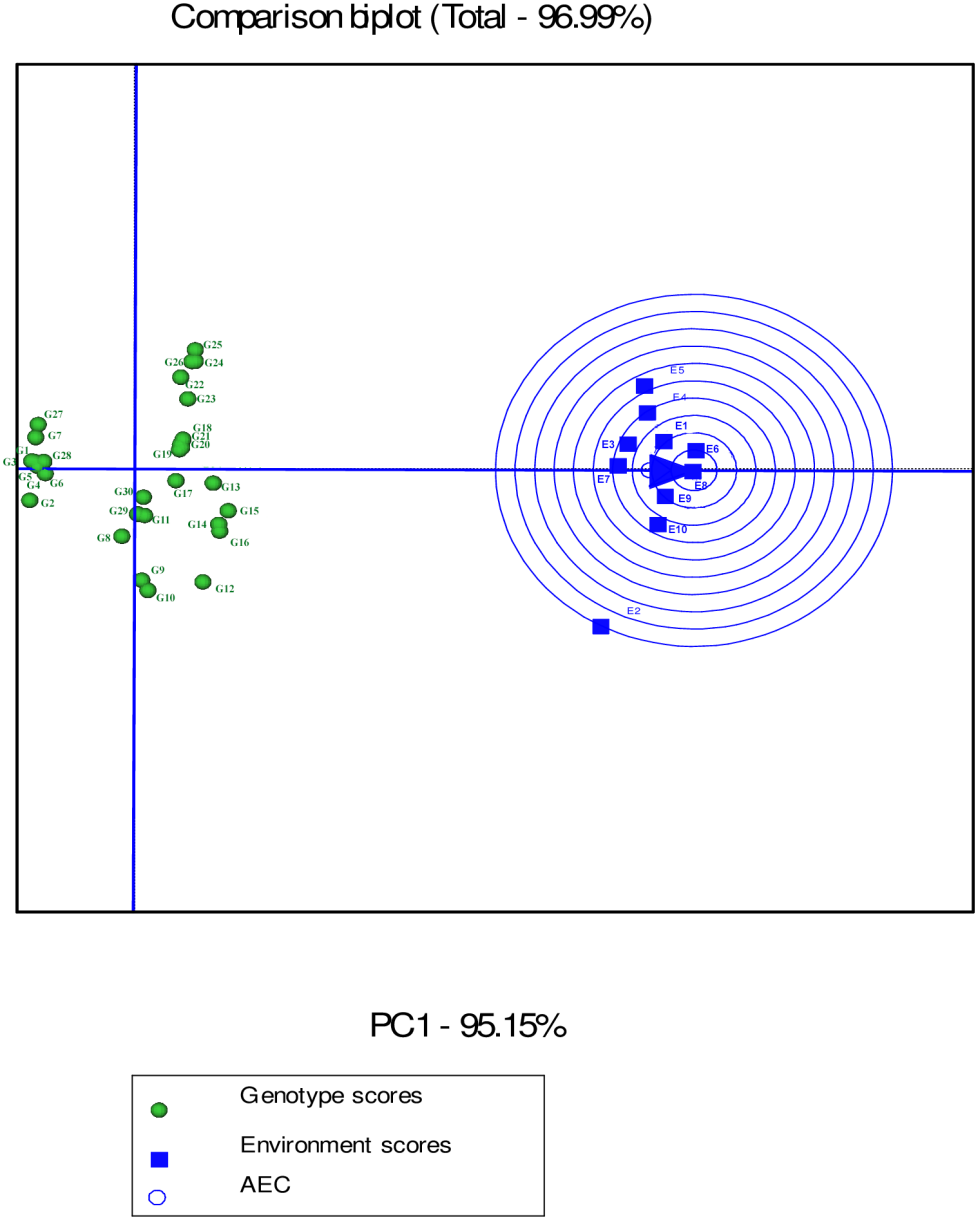
The discrimination and representativeness view of the GGE biplot based on environment-focused scaling for mucilage content of 30 *Plantago* genotypes (*P*. *major*, G1- G7; *P*. *officinalis*, G8-G10; *P*. *amplexicaulis*, G11; *P*. *ovata*, G12-G16; *P*. *psyllium*, G17-G21; *P*. *lanceolata*, G22-G26; *P*. *coronopus*, G27- G28; *P*. *lagopus*, G29- G30) under 10 environments (E) to rank test environments relative to an ideal test environments (represented by center of concentric circles).

Environment-focused scaling GGE biplot shows average environment axis (AEA) and average environment coordination (AEC). The concentric circles on the comparison biplot graph in Figs 10, 11 and 12 help to visualize the distance of environments to AEA, AEC and the biplot origin. The ideal test environment is represented by the center of concentric circles. For seed yield, E10 had smallest angle with AEA (representative) and was near to the center of concentric circles (Fig 10). The GGE biplot showed that E9 and E10 were the most discriminating and representative environments for the evaluation of genotypes based on mucilage yield (Fig 11). E8 and E6 were the best representative and discriminating test environments for evaluation of genotypes for mucilage content (Fig 12).

### Stability, adaptability and performance of genotypes

Genotypic means and rank, SIPC and cultivar superiority (Pi) are displayed in Table 5. Low SICP reflects the stability of genotypes and low GEI [39]. G26 (*P*. *lanceolata*), G16 (*P*. *psyllium*) and G1, G2, G4 and G7 (*P*. *major*) with low SICP scores were the most stable genotypes for seed yield and G7, G1, G3, G4 and G6 (*P*. *major*) for mucilage yield. G4, G5 (*P*. *major*), G8 (*P*. *officinalis*), G13 (*P*. *ovata*) and G18 (*P*. *psyllium*) were the most stable genotypes for mucilage content.

**Table 5:** Mean, habitat mean, sum of interaction principal component (SIPC), AMMI Stability Value (ASV), performance (P_i_) and mean rank of 30 *Plantago* genotypes for seed yield, mucilage yield and mucilage content.

	Seed yield (g m^-2^)						Mucilage yield (g m^-2^)						Mucilage content (%)					
Genotype	Mean	Habitat Mean	SIPC	ASV	P_i_	Mean Rank	Mean	Habitat Mean	SIPC	ASV	P_i_	Mean Rank	Mean	Habitat Mean	SIPC	ASV	P_i_	Mean Rank
G1	23.05	28.56	3.96	4.54	73.77	18.05	1.054	1.267	0.94	0.83	6.441	26.8	4.89	4.3	1.16	0.16	26.86	25.6
G2	22.4	26.14	4.02	2.89	76.17	20.15	0.987	1.102	1.43	1.09	6.597	27.7	4.66	4.73	1.15	0.41	27.84	27.05
G3	23.86	25.11	5.46	4	68.99	16.45	1.081	1.135	0.99	1	6.403	26.8	4.74	4.43	0.98	0.13	27.54	26.7
G4	22.54	25.21	4.1	2.47	74.24	19.4	1.049	1.199	1.03	0.86	6.465	26.9	4.82	5.23	0.68	0.14	27.13	27.1
G5	25.65	24.1	7.1	3.77	53.14	17.5	1.257	1.118	1.65	1.78	6.075	24.2	4.97	4.55	0.71	0.09	26.52	25.9
G6	22.88	26.57	4.58	4.47	76.33	18.6	1.171	1.27	1.13	0.66	6.223	25.6	5.33	5.1	0.98	0.17	24.99	24.1
G7	26.01	30.67	4.17	3.07	53.78	13.05	1.243	1.231	0.93	1.01	6.083	23.9	4.91	4.21	1.24	0.23	26.85	26.2
G8	17.38	14.28	5.93	1.91	115	26.7	1.606	1.824	1.79	0.39	5.392	21.8	9.47	10.1	0.71	0.57	10.94	19.9
G9	18.16	16.59	5.26	2.13	108.05	26.1	1.878	2.326	1.91	0.53	4.879	18.9	10.81	13.19	1.59	0.9	7.9	17.3
G10	17.78	17.28	4.49	1	109.56	26.4	1.925	2.237	1.79	0.17	4.789	17.9	11.2	12.64	1.03	1	6.93	16.2
G11	16.14	22.36	6.39	2.21	125.32	28.3	1.683	1.793	2.4	0.47	5.27	20.8	11.04	10	1.94	0.56	7.73	16.1
G12	40.3	44.62	5.96	3.53	1.75	3.5	6.12	7.139	3.33	1.38	0.285	4.1	15.19	17.21	1.38	0.87	1.12	7.8
G13	42.03	45.73	6.51	4.5	0.23	1.85	6.691	7.368	2.93	0.87	0.071	2.7	15.93	16.37	0.57	0.01	0.51	4.6
G14	38.27	36.7	6.29	2.7	4.68	4.45	6.263	6.92	1.66	0.32	0.196	4.1	16.43	18.6	0.9	0.35	0.37	3.6
G15	41.31	46.69	6.02	2.51	1.27	2.3	7.087	7.954	2.38	0.64	0.013	1.7	17.2	16.82	0.74	0.27	0.06	1.9
G16	40.55	44.92	3.55	3.49	1.44	2.95	6.679	7.444	1.9	0.52	0.098	2.4	16.48	16.34	1.41	0.42	0.36	3.7
G17	32.81	35.34	6.22	5.39	18.08	8.75	4.28	4.425	2.43	2.13	1.541	9.6	13.1	12.52	1.16	0.22	3.57	12.2
G18	32.44	35.74	4.65	4.07	19.07	8.95	4.402	4.669	1.14	0.81	1.383	8	13.59	13.68	0.81	0.35	2.83	11.2
G19	32.17	34.63	5.44	4.06	20.27	9.05	4.282	4.659	2.15	1.33	1.497	9.2	13.38	12.39	1.09	0.3	3.23	12
G20	30.29	31.48	4.26	3.44	27.01	12.35	4.088	4.438	1.92	0.84	1.705	10.3	13.55	11.45	1.46	0.48	3.16	11.2
G21	29.34	33.97	4.5	3.02	31.04	13.65	3.925	4.19	1.9	0.84	1.876	11.4	13.45	11.55	1.34	0.42	3.15	11.2
G22	22.9	33.98	5.26	6.8	78.35	18.75	3.003	4.135	2.31	3.79	3.216	14.1	13.47	12.07	1.63	0.97	3.59	11.3
G23	23.21	33.81	5.95	7.25	77.32	18	3.16	4.526	2.42	4.13	2.998	13.1	14	13.06	1.45	0.8	2.86	9.5
G24	22.83	32.3	5.65	6.23	78.05	19.35	3.322	4.573	2.23	4.51	2.835	11.3	14.55	14.37	1.33	1	2.25	8.1
G25	23.03	33.03	5.21	5.61	74.67	18.35	3.367	4.597	2.67	4.32	2.752	11.5	14.54	13.71	1.54	1.1	2.24	8.2
G26	28.33	34.67	2.51	0.85	37.07	12.75	4.042	4.646	2.65	1.3	1.813	9.8	14.29	15.64	1.76	1	2.55	9.4
G27	27.26	24.5	5.55	4.47	43.38	16	1.371	1.157	1.66	1.7	5.853	23.6	5	5.04	1.15	0.33	26.46	26.5
G28	25.99	22.3	7.91	5.09	52.96	17.25	1.357	1.139	1.64	1.68	5.885	22.5	5.24	5.75	0.91	0.14	25.41	24.85
G29	20.22	22.35	6.63	2.87	87.85	22.55	2.1	2.551	1.97	0.67	4.452	17	10.45	10.94	1.33	0.34	8.59	18.2
G30	19.73	25.03	5.01	1.87	91.24	23.5	2.121	2.705	1.54	0.41	4.409	17.3	10.82	10.58	0.83	0.21	7.65	17.4
*LSD 1%	4.6815	3.264					0.5226	0.728					0.9737	1.397				

*Differences between genotypes means for each trait (column) that are equal to or less than the LSD 1% is not significant.

Low SICP and ASV reflect the stability of genotypes and low GE interaction. The smaller Pi and mean rank the smaller distance to the genotype with maximum yield and shows best performance.

P_i_ is the mean squares of distance between genotypes i and ‘i’ where ‘i’ is the genotype with maximum response over all trials [33]. Genotypes with smaller Pi are closer to the genotype with the maximum yield. Pi represents genotypic superiority in the sense of general adaptability or wide adaptation. The mean rank of each genotype based on P_i_ scores in all environments is presented in Table 5. Based on Pi and mean rank, *P*. *ovata* and *P*. *psylilum* were the best for seed and mucilage yield, whilst *P*. *ovata* and *P*. *lanceolata* were the best for mucilage content.

The results of the AMMI analysis showed that G12, G13, G14, G15 and G16 (*P*. *ovata*) had the best performance for seed and mucilage yield in all environments. G12, G13, G14, G15 and G16 (*P*. *ovata*), G20 (*P*. *psyllium*), and G22, G23, G24, G25 and G26 (*P*. *lanceolata*) were superior genotypes for mucilage content (Table 4). Genotypes and environments with the same IPCA1 sign have positive interactions, but different signs show negative interactions. The AMMI1 biplot graph for seed yield (Fig 1; S1 Table) showed that genotypes with negative IPCA1 including G11 (*P*. *ampleexicaulis*), G8, G9 and G10 (*P*. *officinalis*), G1, G2, G3, G4, G6 and G7 (*P*. *major*), G22, G23, G24 and G25 (*P*. *lanceolata*) had positive interactions with normal irrigation conditions (E1, E3, E5, E7 and E9) and negative interactions with drought conditions (E2, E4, E6, E8 and E10) for seed yield. G12, G13, G14, G15 and G16 (*P*. *ovata*), G17, G18, G19, G20 and G21 (*P*. *psyllium*), G26 (*P*. *lanceolata*), G5 (*P*. *major*), G27 and G28 (*P*. *coronopus*) and G29 and G30 (*P*. *lagopus*) with positive IPCA1 had positive interactions with drought conditions and negative interactions with normal irrigation conditions. Accordingly, these genotypes could be regarded as candidates for plant selection based on seed yield under drought conditions. G10 (*P*. *officinalis*) with low seed yield (17.78 g m^-2^) and G26 (*P*. *lanceolata*) with moderate seed yield (28.33 g m^-2^) were stable genotypes. G13, G16, G12, G14 and G15 (*P*. *ovata*) showed the highest mean seed yield and they were well adapted to drought stress conditions. Analysis of the AMMI1 biplot for mucilage yield indicated that G12, G13, G14, G15 and G16 (*P*. *ovata*), G18, G17, G19, G20 and G21 (*P*. *psyllium*) had the highest mucilage yield. In addition, G12, G13, G14, G15 and G16 (*P*. *ovata*), G18, G17, G19, G20 and G21 (*P*. *psyllium*), G29 and G30 (*P*. *lagopus*), G27 and G28 (*P*. *coronopus*) and G1, G2, G3, G4, G5, G6 and G7 (*P*. *major*) had positive interactions with drought conditions for mucilage yield (Fig 2; S2 Table). G26 (*P*. *lanceolata*) with high mucilage yield and with positive interactions with normal conditions could be a good candidate for cultivation and domestication under well-irrigated conditions. The AMMI1 biplot based on mucilage yield showed that G22, G23, G24 and G25 (*P*. *lanceolata*) were the best in E5 (Fig 2). G12, G13, G14, G15 and G16 (*P*.*ovata*), G17, G18, G19, G20 and G21 (*P*. *psyllium*) and G22, G23, G24, G25 and G26 (*P*. *lanceolata*) had the highest mucilage content (Fig 3; S3 Table).

The AMMI2 biplot graph showed that *P*. *lanceolata* (G26), *P*. *officinalis* (G10), *P*. *ovata* (G14), *P*. *amplexcaulis* (G11) and *P*. *major* (G4) were the most stable genotypes for seed yield (Fig 4; S1 Table). G5 (*P*. *major*) and G27 and G28 (*P*. *coronopus*) showed special adaptability to drought conditions for seed yield in Shiraz trials (E2, E5 and E7) (Fig 4). G12, G13, G14, G15 and G16 (*P*. *ovata*), G18, G19, G20 and G21 (*P*. *psyllium*), G26 (*P*. *lanceolata*), and G29 and G30 (*P*. *lagopus*) were drought adapted genotypes for seed yield in Kooshkak (E8 and E10). G22, G23, G24 and G25 (*P*. *lanceolata*) were appropriate genotypes for seed yield in Kooshkak under normal irrigation conditions (E7 and E9) (Fig 4). G8, G9 and G10 (*P*. *officinalis*), G11 (*P*. *amplexicaulis*) and G2, G3, G4, G6 and G7 (*P*. *major*) showed special adaption to normal irrigation conditions in Shiraz (E1, E3 and E5). G5 and G26 had different responses to drought stress conditions compared with other genotypes of *P*. *major* and *P*. *lanceloata*. This showed the existence of interspecific variation in *Plantago* genotypes in dealing with drought stress conditions.

Based on the AMMI2 biplot, G1 (*P*. *major*), G9 and G10 (*P*. *officinalis*), G18, G20 and G21 (*P*. *psyllium*) and G22, G24, G25 and G26 (*P*. *lanceolata*) were the most stable genotypes for mucilage yield (Fig 5; S2 Table). G12, G13, G14, G15, and G16 (*P*. *ovata*), G17, G19 and G20 (*P*. *psyllium*) and G29 and G30 (*P*. *lagopus*) showed adaptability to drought stress conditions for mucilage yield in Kooshkak (Fig 5). G1, G2, G3, G4, G5, G6 and G7 (*P*. *major*), G11 (*P*. *amplexicaulis*) and G27 and G28 (*P*. *coronopus*) showed special adaptability to drought conditions (E2, E4 and E6) for mucilage yield. G22, G24, G25 and G26 (*P*. *lanceolata*) and G9 (*P*. *officinalis*) showed adaptability to normal irrigation conditions (E1, E3 and E7) (Fig 5). G23 (*P*. *lanceolata*) showed special adaptability to normal irrigation conditions (E7 and E9) for mucilage yield in Kooshkak (Fig 5).

The AMMI2 analysis showed that G5 and G6 (*P*. *major*) and G13 (*P*. *ovata*) were most stable genotypes for mucilage content (Fig 6; S3 Table). *P*. *amplexicaulis* (G11) which was adaptable to normal irrigation conditions (E1, E7 and E5) for seed yield, had special adaptability to drought conditions (E6 and E6) for mucilage content.

The “which-won-where” GGE biplot for seed yield (Fig 7) and mucilage yield (Fig 8) placed G12, G13, G14, G15 and G16 (*P*. *ovata*) and G17, G18, G19, G26 (*P*. *lanceolata*) and G20 (*P*. *psyllium*) in the sector of drought environments. These results indicated that such genotypes were well adapted to drought conditions for seed yield. G26 (*P*. *lanceolata*) was located near the place of normal environments in the “which-won-where” GGE biplot for mucilage yield (Fig 8). The place of genotypes on the vertices of the polygon in the “which-won-where” biplot shows the best or the poorest performance of each genotype [32]. The perpendicular of the polygon facilitates visual comparison of the distance between genotypes and environments [32]. The place of genotypes and environments in the “which-won-where” GGE biplot for mucilage content differed from those of mucilage and seed yield showing a different response of mucilage content to genotypic and environmental variations. This result agreed with the results of AMMI1 (Fig 3).

**Fig 13:**
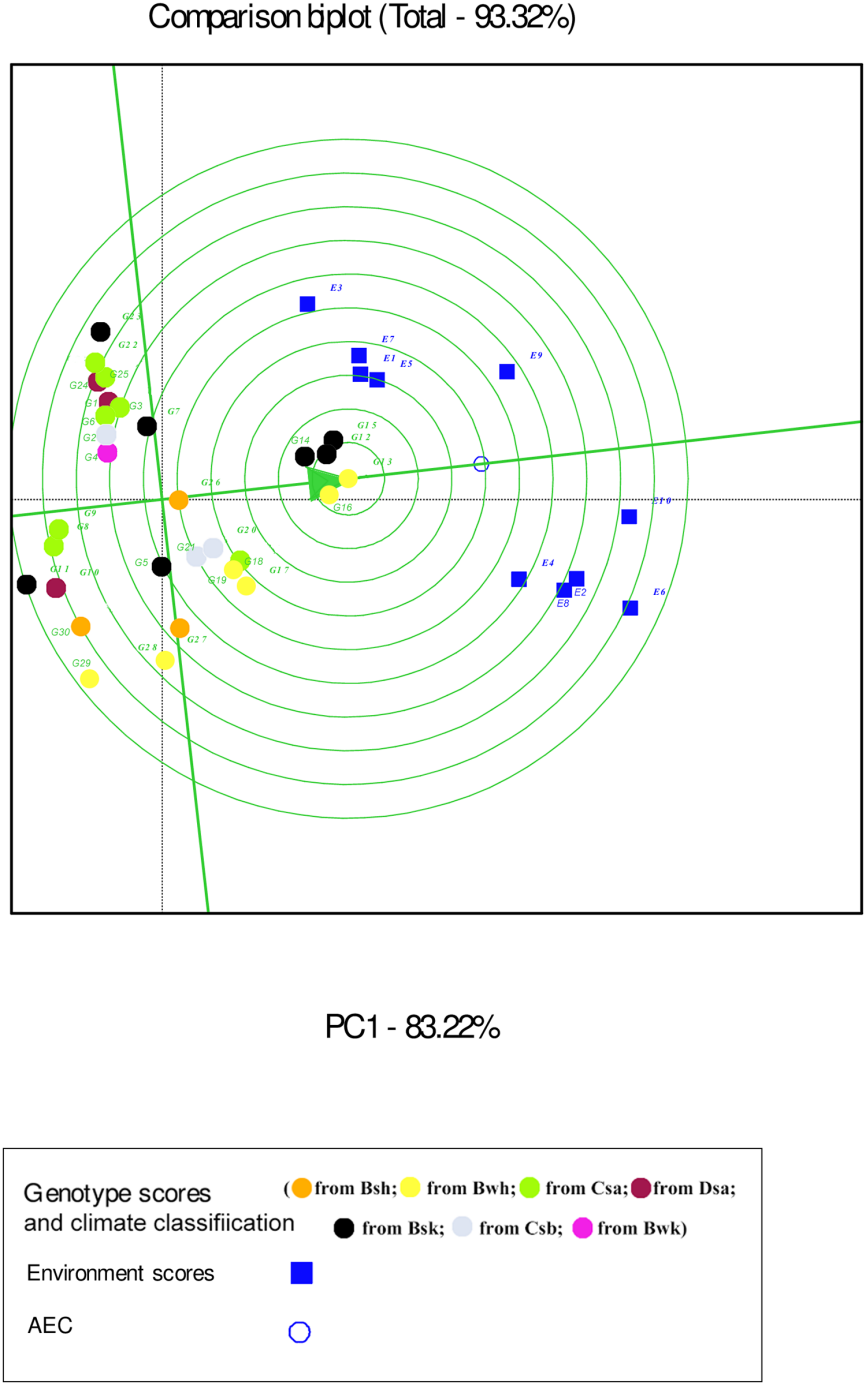
The average environment coordination (AEC) view of GGE biplot based on genotype-focused scaling for seed yield if 30 *Plantago* genotypes (*P*. *major*, G1- G7; *P*. *officinalis*, G8-G10; *P*. *amplexicaulis*, G11; *P*. *ovata*, G12-G16; *P*. *psyllium*, G17-G21; *P*. *lanceolata*, G22-G26; *P*. *coronopus*, G27- G28; *P*. *lagopus*, G29- G30) under 10 environments (E) to rank genotypes relative to an ideal genotype (The center of the concentric circles). Köppen-Geiger climate classification [45] letter: B: arid, s: summer dry, k: cold arid.

**Fig 14:**
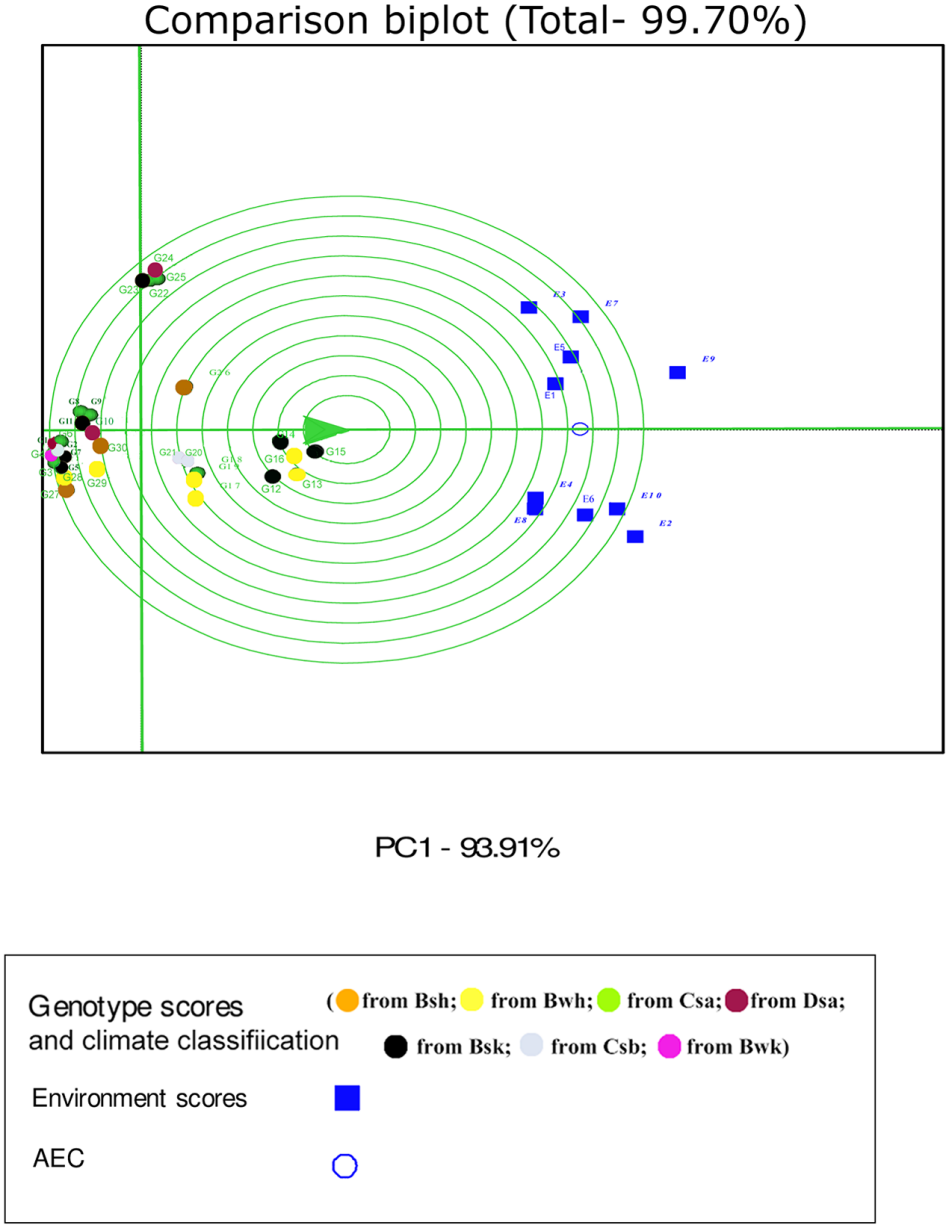
The average environment coordination (AEC) view of GGE biplot based on genotype-focused scaling for mucilage content of 30 *Plantago* genotypes (*P*. *major*, G1- G7; *P*. *officinalis*, G8-G10; *P*. *amplexicaulis*, G11; *P*. *ovata*, G12-G16; *P*. *psyllium*, G17-G21; *P*. *lanceolata*, G22-G26; *P*. *coronopus*, G27- G28; *P*. *lagopus*, G29- G30) under 10 environments (E) to rank genotypes relative to an ideal genotype (The center of the concentric circles). Köppen-Geiger climate classification [45] letter: B: arid, s: summer dry, k: cold arid.

**Fig 15:**
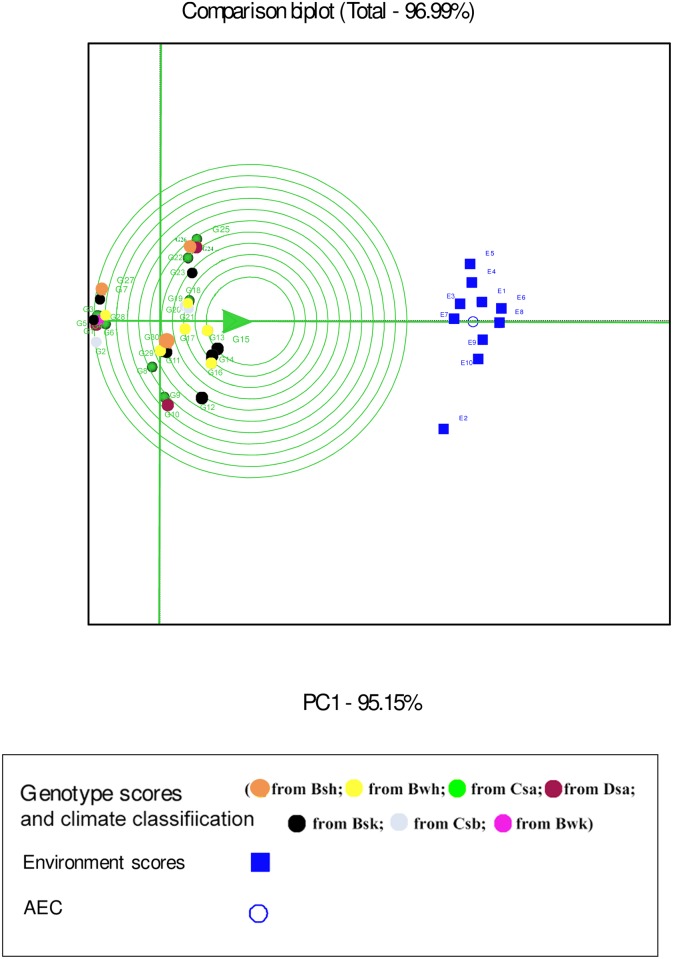
The average environment coordination (AEC) view of GGE biplot based on genotype-focused scaling for mucilage content of 30 *Plantago* genotypes (*P*. *major*, G1- G7; *P*. *officinalis*, G8-G10; *P*. *amplexicaulis*, G11; *P*. *ovata*, G12-G16; *P*. *psyllium*, G17-G21; *P*. *lanceolata*, G22-G26; *P*. *coronopus*, G27- G28; *P*. *lagopus*, G29- G30) under 10 environments (E) to rank genotypes relative to an ideal genotype (The center of the concentric circles). Köppen-Geiger climate classification [45] letter: B: arid, s: summer dry, k: cold arid.

The GGE biplot of genotype-focused scaling showed that *P*. *ovata* and *P*. *psyllium* were at the top of the rankings for seed yield, mucilage yield and content and were closer to the place of the ideal genotype in the biplot graph (Figs 13, 14 and 15; S4 Table). In the graphical analysis of IPCAs, the first principal component (IPCA1) represents cultivar productivity, and the second is associated with cultivar stability [32]. Hence, the GGE biplot showed that the ideal genotype must have a high IPCA1 value (high productivity) and an IPCA2 value close to zero (more stable). The ideal genotype is located in the center of concentric circles of Figs 13, 14 and 15. The genotypes G13 (*P*. *ovata*) and G26 (*P*. *lanceolata*), for seed yield, G9, G10, G11, G8 (*P*. *officinalis*) and G14 (*P*. *ovata*) for mucilage yield, and G1, G3, G4, G5 and G6 (*P*. *major*), G28 (*P*. *coronopus*) for mucilage content were the most stable. The position of these genotypes was in close proximity to the stability axis (average environment axis (AEA)) (Figs 13, 14 and 15). Stable genotypes are desirable only when they have high mean performances and are located closer to the place of ideal genotype in GGE biplot [32].

The response of G5 and G7 (*P*. *major*), and G26 (*P*. *lanceolata*) to GEI for seed yield (Figs 7, 10 and 13) and G26 (*P*. *lanceolata*) for seed and mucilage yield (Figs 8, 11 and 14) reflects the existence of the interspecific variation being used in *Plantago* breeding.

## Discussion

In the present study, the response of thirty *Plantago* genotypes to environmental conditions was investigated by the AMMI and GGE biplot models and the simple statistic of Pi on the basis of variations in mucilage content and seed yield traits. In the present study, significant differences were found between *Plantago* species for genotypic means and ranks, SIPC and cultivar superiority (Pi) for mucilage and seed yield. Variation in the interspecific P_i_ index shows the existence of genetic diversity that can be exploited in breeding programs of *Plantago*. Genetic variation is the fundamental basis for the improvement of *Plantago* [1]. Results by Canter et al. [1] indicated exploitation of the genetic potential of medicinal plants is still in its initial stages but variation exists between medicinal plants for breeding. The results of the present study revealed that mucilage content was not significantly different in drought versus irrigated conditions, although slight increases in content were observed in some drought environments. It has been reported that drought enhances secondary metabolites due to a stress-related decline in biomass production associated with an unchanged biosynthesis rate of natural products or an authentic enhancement of the total secondary metabolite content [10, 15, 17–20, 54]. The results showed that the effect of genotype on mucilage yield and content was more salient than on seed yield whilst seed yield slightly had more response to environmental variations.

AMMI analysis of variance indicated that genotype was the major contributor to the total variation in mucilage and seed yield in comparison to environment and genotype × environment interactions. In a study on rice, genotype accounted for 67.11% of the total variation of grain yield [53]. Similarly, Islam et al. [55] reported that 57.34% of the total sum of squares rice grain yield was attributed to the effect of genotype. Contrary to these results, analysis of yield stability in yellow passion fruit varieties indicated that 61% of the total sum of squares was explained by the environment and 5% and 34% were attributable to the genotype and GEI effects, respectively [52]. One may propose a “70-20-10” saying that a median yield trial has about 70% of variation in E, 20% in GEI and 10% in G, but as the genotypes (in contrast to similar cultivars) and environments become more diverse the share of G and GEI tends to increase [56, 57]. Accordingly, for the purpose of plant selection, the rankings of genotypes matter, which are determined by G and GEI and in such cases, reducing or eliminating the influence of environmental main effects is interested [35, 58]. Our study indicated that the contribution of AMMI2 to GEI sum of squares was in agreement with the share of AMMI2 in total variance in Jamshidmoghaddam and Pourdad [39] and with the results of a study by Oliveira et al. [55] who showed that AMMI2 biplot may be more accurate to extract GEI variation given as it contains information of two IPCAs and greater pattern proportion compared to the AMMI1 which considers only the IPCA1. The AMMI2 model was simple, allowing conclusions to be made about stability, genotypic performance, genetic divergence between cultivars, and the environments that optimize cultivar performance [59]. In the AMMI2 biplot graph, close genotypes and environments have positive associations and the place of stable genotypes is near the origin of biplot [53, 59, 60]. In the present study, AMMI2 showed some of genotypes (i.e. G10, G11 and G26) were adapted to drought stress conditions in view of mucilage yield or content whilst they had variations for seed yield. This result shows different response of mucilage as a secondary metabolite versus seed yield to drought stress conditions [61].

The perpendicular of the polygon in the “which-won-where” GGE biplot facilitates the visual comparison of distance between genotypes and environments and helps identify the representativeness of environments and their discriminating ability [32, 33]. In the present study, seed yield was mostly affected by the irrigation regime and studied environments were divided into two meaningful mega- environments on the basis of drought conditions. In a study on eighteen wheat genotypes, nine environments were classified into four clusters based on discriminating ability of genotypes and representativeness of environments [33]. Akter et al. [53] identified the most discriminating environments for grain yield using the AMMI analysis of twelve rice genotypes and five environments in Bangladesh. Identifying mega-environments helps evaluate the discriminating ability and representativeness of the environments with a view to detect locations that can be culled without losing important information about the genotypes [62]. In this study, E9 (normal irrigation condition in Kooshkak) was grouped with drought condition trials. This was possibly because of the low precipitation in 2014 in Kooshkak (Table 2). The information on mega- environments allows breeders to identify discriminating and representative environments that are good test environments for detection of generally adapted genotypes or breeding for adaptation to specific environmental factors [22, 62]. In addition, by adding mega-environment boundaries, breeders can determine whether a test location is predictive for a given environment or else is frequently crossing mega-environment boundaries from year to year [63]. The GGE biplot analysis for mucilage content showed that this trait was discriminated approximately by the year effect. This result showed that environmental conditions cause noticeable effects on the life cycles of *Plantago* that was in agreement with the results of previous studies on medicinal plants [16, 17, 25, 64].

In the GGE biplot graphs, the smallest angle with AEA represents the most representative environment [32]. The most informative and discriminating environment is the farthest from the origin of biplot, whereas a non-discriminating environment which plotted near the origin is not informative, provides little information and should not be used as a test environment in plant breeding programs aimed at cultivar release [32, 39]. Discriminating and non-representative environments are candidate test environments to identify genotypes with special adaptability, whilst discriminating and representative environments are good test environments to verify broadly adapted genotypes. Environment-focused scaling GGE biplot revealed that E10 for seed yield and E9 and E10 for mucilage yield were the most discriminating and representative environments. A test environment can be characterized by its similarity with other environments and its discrimination power [65]. The results in the present study showed that drought condition was discriminating for specially adapted genotypes based on seed yield although it was not representative. The results of GGE biplot analysis agreed with previous reports on mucilage content showing it was more responsive to environmental conditions in comparison to seed yield in *Plantago* [1, 25, 53, 66].

The AMMI model has been shown to be effective as it contributed a large portion of the GEI sum of squares and separating the main and interaction effects. The GGE biplot model is effective for identifying the stable cultivars across environments and identifying the best cultivars for mega-environment differentiation [63, 67]. The results showed that the AMMI and GGE biplot models had similar results in view of specific adaptability to environmental conditions. Nevertheless, contrary results were obtained for environmental contribution to the stability of genotypes. For instance, AMMI1 introduced E7 with low contribution to the GE interaction for seed yield and E1 for mucilage yield, but in the AMMI2 and GGE biplot analyses E7 and E1 were high interactive for seed and mucilage yield, respectively. In AMMI2, all environments had high contribution to the GE interaction for seed yield, whilst in GGE biplot E3, E1, E7 and E5 were found as relatively low discriminating with high contribution to genotype stability for seed yield. In AMMI1, stable genotypes were detected only based on IPCA1 scores but relatively different results were found in the AMMI2 and GGE biplot models due to contribution of two IPCAs information in detection of stable genotypes [52, 53, 55]. On the other hand, the different statistical basis of IPCA in AMMI2 and PC in GGE biplot leads to some differences in stability results [59]. Although differences in the biplot view of genotypic stability were found, the results of AMMI1, AMMI2 and GGE biplot analyses agreed for some of genotypes. For instance, G26 (*P*. *lanceolata*) was detected as stable genotype for seed yield based on AMMI1, AMMI2 and GGE biplot. *P*. *ovata*, *P*. *psyllium*, *P*. *lagopus* and *P*. *coronopus* were identified as drought- adapted species in AMMI and GGE biplot analyses. The AMMI1, AMMI2 and GGE biplot models also showed similar results for adaptability of genotypes based on mucilage yield and content. Furthermore, the position of G5 (*P*. *major*) was relatively similar in AMMI1, AMMI2 and GGE biplot graphs. Also, the distribution of genotypes in both AMMI graphs and GGE biplots with respect to the traits tested demonstrated that genotypes originated from habitats having similar environmental conditions placed close each other. This may demonstrate genotypic characteristics typical for their habitats. This might be due to the effects of habitation variations of a trait in a species which was in accord with the results of Wolff and Delden [68] who reported variations in *Plantago lanceolata* in the natural habitats was also observed in the greenhouse and experimental garden. Results of the same study revealed that inhomogeneous habitat characteristics and level of phenotypic plasticity affects genetic variation and species microevolution. In a study on wheat, with respect to grain yield, the genotype rankings in the GGE biplot was significantly correlated with the rankings identified in AMMI analysis [46]. In the present study, the results of P_i_ statistic agreed with the results of the AMMI1, AMMI2 and GGE biplot models about top ranked- genotypes in all environments.

## Conclusion

Mean seed yield was highly affected by genotype variation and environmental conditions, while mucilage as a secondary metabolite was mostly affected by the effect of genotype. AMMI stability analysis showed that environments divided into normal and drought conditions for mean seed yield. In the AMMI1 biplot, distribution of environments for mucilage content was mostly based on location and the year of experiment. Environments were divided into two meaningful mega- environments for seed yield based on drought conditions. Drought cannot discriminate genotypes for mucilage yield and content, therefore other environmental conditions may be responsible for this purpose. E10 for seed yield and E9 for mucilage yield and content were the best representative environments with highest ability discriminating of genotypes. *P*. *ovata* and *P*. *pysllium* with the highest seed yield showed positive interaction with drought conditions for seed yield and therefore are good candidates for cultivation and domestication under arid climate of Iran. *P*. *coronopus* and *P*. *lagopus* also showed positive interaction with drought stress conditions, but they had moderately low mean seed yield. *P*. *major*, *P*. *officinalis*, *P*. *amplexicaulis* and *P*. *lanceolata* showed positive interaction with normal irrigation conditions and negative interaction with drought conditions for mean seed yield. The origin of the genotypes G5 and G7 (*P*. *major*), G14 and G15 (*P*. *ovata*), the identified stable genotypes, belongs to the Bsk climate (arid, summer dry and cold arid) which is similar to the climate classification of the experimental field in the present study. G26 (*P*. *lanceolata*) was another useful genotype with respect to the traits tested in this study. This genotype was collected from a climate (the Bsh: arid, summer dry and hot arid) being relatively similar to the climate of the experimental field. The meteorological data indicated that *Plantago* could grow in wide geographical ranges differing in altitudes, annual rainfall and temperature. These show the importance of climate in cultivation and domestication of *Plantago*. Results showed that adaptability of some genotypes for mucilage yield and content was different from seed yield. This result emphasizes different response of secondary metabolite and seed yield to stress conditions. Overall, sufficient inter and intra specific variations were found between *Plantago* species laying the foundation for plant selection and improvement of seed yield, mucilage yield and content in breeding programs aimed at production of new cultivars.

## Supporting information

S1 Table Means and IPCA scores of environments and genotypes used in AMMI1 and AMMI2 analyses and figures for seed yield (E and e: Environment; G and g: genotype; M: mean). (XLSX)

S2 Table Means and IPCA scores of environments and genotypes used in AMMI1 and AMMI2 analyses and figures for mucilage yield (E and e: Environment; G and g: genotype; m: mean). (XLSX)

S3 Table Means and IPCA scores of environments and genotypes used in AMMI1 and AMMI2 analyses and figures for mucilage content (E and e: Environment; G and g: genotype; m: mean). (XLSX)

S4 Table The template matrix for mean seed yield, mucilage yield and content matrix for GGE biplot analyses. (XLSX)

## References

[1] Canter PH, Thomas H, Ernst E. Bringing medicinal plants into cultivation: opportunities and challenges for biotechnology. Trends Biotechnol. 2005; 23(4):180–5. doi: 10.1016/j.tibtech.2005.02.002 15780709 10.1016/j.tibtech.2005.02.002

[2] Ahvazi M, Khalighi-Sigaroodi F, Charkhchiyan MM, Mojab F, Mozaffarian VA, Zakeri H. Introduction of medicinal plants species with the most traditional usage in Alamut region. Iran J Pharm Res. 2011; 20: 11(1):185–194 PMC3813099 24250441

[3] Bekele E. Study on actual situation of medicinal plants in Ethiopia, Japan Association for International Collaboration of Agriculture and Forestry. pp 54–60. 2007.

[4] Shahriari Z, Heidari B. Response to drought stress and karyotype analysis in *Plantago ovata* 2^nd^ International and 14^th^ Iranian Genetic Congress. Rasht University, Rasht, Iran, 2016.

[5] Koocheki A, Tabrizi L, NassiriMahallati M. Organic cultivation of *Plantago ovata* and *Plantago psyllium* in response to water stress. J Iranian Field Crop Res. 2004; 2(1): 67–78.

[6] Koocheki A, Tabrizi L, NassiriMahallati M. The effect of irrigation intervals and manure on quantitative and qualitative characteristics of *Plantago ovata* and *Plantago psyllium*. Asian J Plant Sci. 2007; 6(8): 1229–1234.

[7] Noorhosseini Niyaki SA, Ashori Latmahalleh D, Allahyari MS, Doozane Masooleh P. Socio-economic factors for adoption of medicinal plants cultivation in Eshkevarat region, north of Iran. J Med Plants Res. 2011; 5: 30–38.

[8] Zubair M. Genetic variation, biochemical contents and wound healing activity of Plantago major. Dissertation, Acta Universitatis Agriculturae, Sueciae. 2012.

[9] Zubair M, Nybom H, Ahnlund M, Rumpunen K. Detection of genetic and phytochemical differences between and within populations of *Plantago major* L. (plantain). Sci Hort. 2012; 136:9–16.

[10] Walter J, Kreyling J, Singh BK, Jentsch A. Effects of extreme weather events and legume presence on mycorrhization of *Plantago lanceolata* and *Holcus lanatus* in the field. Plant Biol. 2016; 18(2):262–70. doi: 10.1111/plb.12379 26284575 10.1111/plb.12379

[11] Parsaei P, Bahmani M, Naghdi N, Asadi-Samani M, Rafieian-Kopaei M. The most important medicinal plants effective on constipation by the ethnobotanical documents in Iran: A review. Der Pharm Lett 8(2), 2016.

[12] Haddadian K, Haddadian K, Zahmatkash M. A review of *Plantago* plant. Int J Trad Knowl. 2014; 13(4):681–685.

[13] Mirmasumi M, Ebrahimzadeh H, Tabatabaei SMF. Mucilage production in tissue culture of *Plantago lanceolata*. J Agric Sci Technol. 2001; 3: 155–160.

[14] Hanley ME. The accumulation of the stress metabolite praline in Plantago lanceolata as a response to lead pollution, Durham theses, Durham University. 1990.

[15] Thomas CD, Cameron A, Green RE, Bakkenes M, Beaumont LJ, Collingham YC, et al Extinction risk from climate change. Nature 2004; 427:145–148. doi: 10.1038/nature02121 14712274 10.1038/nature02121

[16] Malcolm JR, Liu C, Neilson RP, Hansen L, Hannah L. Global warming and extinctions of endemic species from biodiversity hotspots. Biol Conserv. 2006; 20(2):538–548. 10.1111/j.1523-1739.2006.00364.x 16903114

[17] Cavaliere C. The effects of climate change on medicinal and aromatic plants. HerbalGram 81:44–57.

[18] Selmar D, Kleinwächter M. Stress enhances the synthesis of secondary plant products: The impact of stress-related over-reduction on the accumulation of natural products. Plant Cell Physiol. 2013; 54(6): 817–826. doi: 10.1093/pcp/pct054 23612932 10.1093/pcp/pct054

[19] Selmar D, Kleinwächter M. Influencing the product quality by deliberately applying drought stress during the cultivation of medicinal plants. Ind Crops Prod. 2013; 42:558–566.

[20] Van De Velde H, Bonte D, AbdElgawad H, Asard H, Nijs I. Combined elevated CO_2_ and climate warming induces lagged effects of drought in *Lolium perenne* and *Plantago lanceolata*. Plant Ecology. 2015; 216(8): 1047–59.

[21] Fougat RS, Joshi C, Kulkarni K, Kumar S, Patel A, Sakure A, et al Rapid development of microsatellite markers for *Plantago ovata* Forsk: using next generation sequencing and their cross-species transferability. Agriculture 2014; 2: 199–216.

[22] Xu Y. Envirotyping for deciphering environmental impacts on crop plants. Theor Appl Genet. 2016; 129: 653–673. doi: 10.1007/s00122-016-2691-5 26932121 10.1007/s00122-016-2691-5 PMC4799247

[23] Simmonds NW. Selection for local adaptation in a plant breeding programme. Theor Appl Genet. 1991; 82:363–367 24213182 10.1007/BF02190624

[24] Carrubba, A, Torre A R, Matranga A. Cultivation trials of some aromatic and medicinal plants in a semi-arid mediterranean environment. Proceeding of an International Conference on MAP. Acta Hortic. 2002; 576: 207–213.

[25] Mathur S, Sharma S, Gupta MM, Kumar S. Evaluation of an Indian germplasm collection of the medicinal plant *Bacopa monnieri* by use of multivariate approaches. Euphytica 2003; 133:255–265.

[26] Pank F. Breeding of Medicinal Plants, in Medicinal Plant Biotechnology: From Basic Research to Industrial Applications (eds Kayser O. and Quax W. J.), Wiley-VCH Verlag GmbH, Weinheim, Germany 2006.

[27] Aghaei K, Komatsu S. Crop and medicinal plants proteomics in response to salt stress. Front Plant Sci. 2013; 31: 4–8. 10.3389/fpls.2013.00008 PMC3560237 23386857

[28] Ghaed-Rahimi L, Heidari B, Dadkhodaie A. Genotype × environment interactions for wheat grain yield and antioxidant changes in association with drought stress. Archives Agron Soil Sci. 2013; 61(2): 1531–171.

[29] Al Hassan M, Pacurar A, López-Gresa MP, Donat-Torres MP, Llinares JV, Boscaiu M, et al Effects of salt stress on three ecologically distinct *Plantago* species. PLoS ONE 2016; 11(8). 10.1371/journal.pone.0160236 PMC4973956 27490924

[30] Balcha A. Medicinal plants used in traditional medicine by Oromo people, Ghimbi District, Southwest Ethiopia. J Ethnobiol Ethnomed. 2014; 10:40 doi: 10.1186/1746-4269-10-40 24885586 10.1186/1746-4269-10-40 PMC4060869

[31] Yan W, Tinker NA. An integrated biplot analysis system for displaying, interpreting, and exploring genotype by environment interactions. Crop Sci. 2005; 45: 1004–1016.

[32] Yan W, Tinker AN. Biplot analysis of multi-environment trial data: Principles and applications. Can J Plant Sci. 2006; 86(3): 623–645.

[33] Lin CS, Binns MR. A. superiority measure of cultivar performance for cultivar × location data. Can J Plant Sci. 1988; 68: 193–198.

[34] Lin CS, Binns MR. Concepts and methods for analyzing regional trial data for cultivar and location selection. Plant Breed Rev. 1994; 12: 271–297.

[35] Gauch HG. Statistical analysis of regional yield trials: AMMI analysis of factorial designs, Elsevier Health Sciences, Amsterdam, Netherlands, 1992.

[36] Annicchiarico P. Joint regression vs AMMI analysis of genotype-environment interactions for cereals in Italy. Euphytica 1997; 94: 53–62.

[37] Gauch HG. Model selection and validation for yield trials with interaction. Biometrics 1988; 44: 705–715

[38] Moreno-González J, Crossa J, Cornelius PL. Genotype × environment interaction in multi-environment trials using shrinkage factors for AMMI models. Euphytica 2004; 137: 119–127.

[39] Jamshidmoghaddam M, Pourdad SS. Genotype × Environment interactions for seed yield in rainfed winter safflower (*Carthamus tinctorius* L.) multi-environment trials in Iran. Euphytica 2013; 190: 357–369.

[40] Hongyu K, García-Peña M, Borges de Araújo L, Tadeu dos Santos Dias C. Statistical analysis of yield trials by AMMI analysis of genotype × environment interaction. Biom Lett. 2014; 51(2): 89–102.

[41] Latzel V, Ha´jek T, Klimesˇova´ J, Go´mez S. Nutrients and disturbance history in two *Plantago* species: maternal effects as a clue for observed dichotomy between re sprouting and seeding strategies. Oikos 2009; 118: 1669–1678.

[42] Rahimi A, Sayadi F, Dashti H, Tajabadi Pour A. Effects of water and nitrogen supply on growth, water-use efficiency and mucilage yield of isabgol (*Plantago ovata* Forsk). J Soil Sci Plant Nutr. 2013; 13 (2): 341–354.

[43] Sayadi F, Rahimi A, Dashti H, Tajabadi Pour A. Influence of drought stress and nitrogen on dry matter partitioning of Isabgul (*Plantago ovata* Forsk). Iranian J Medic Arom Plants 2013; 29(4):783–94.

[44] Miehe-Steier A, Roscher C, Reichelt M, Gershenzon J, Unsicker SB. Light and nutrient dependent responses in secondary metabolites of *Plantago lanceolata* offspring are due to phenotypic plasticity in experimental grasslands. PLoS ONE 2015; 10 (9). 10.1371/journal.pone.0136073 PMC4559451 26336100

[45] Kottek M, Grieser J, Beck C, Rudolf B, Rubel F. World Map of the Köppen-Geiger climate classification updated. Meteorol Z. 2006; 15: 259–263.

[46] Sharma PK, Koul AK. Mucilage in seeds of *Plantago ovata* and its wild allies. J Ethnopharmacol. 1986; 17:289–95. 3807392 10.1016/0378-8741(86)90118-2

[47] Zobel RW, Wright MJ, Gauch HG. Statistical analysis of a yield trials. Agron J 1988; 80:388–393.

[48] Purchase JL, Hatting H, Van Deventer CS. Genotype × environment interaction of winter wheat in South Africa: II. Stability analysis of yield performance. South Afric J Plant Soil 2000; 17:101–107.

[49] Sneller CH, Kilgore-Norquest L, Dombek D. Repeatability of yield stability statistics in soybean. Crop Sci 1997; 37:383–390.

[50] Yan W. Singular value partitioning for biplot analysis of multi-environment trial data. Agron J. 2002; 94: 990–996.

[51] GENSTAT. GENSTAT 12th edn. VSN International Ltd (VSNi), Hertfordshire. 2009. http://www.vsni.co.uk

[52] Oliveira EJ, de Freitas JPX, de Jesus ON. AMMI analysis of the adaptability and yield stability of yellow passion fruit varieties. Sci Agric. 2009; 71(2): 139–145.

[53] Akter A, Jamil Hassan M, Umma Kulsum M, Islam MR, Hossain K. AMMI biplot analysis for stability of grain yield in hybrid rice (*Oryza sativa*). J Rice Res. 2014; 2(2): 126–129.

[54] Niinemets Ü Uncovering the hidden facets of drought stress: secondary metabolites make the difference. Tree Physiol. 2015; 36 (2): 129–132. doi: 10.1093/treephys/tpv128 26687175 10.1093/treephys/tpv128

[55] Islam MR, Anisuzzaman M, Khatun H, Sharma N, Islam MZ, Akter A, et al AMMI analysis of yield performance and stability of rice genotypes across different haor areas. Eco-friendly Agril J. 2014; 7(02): 20–24.

[56] Romagosa IPN, Fox LF, Garcia del Moral JM, Ramos B, Garcia del Moral F, Rocade T, et al Integration of statistical and physiological analyses of adaptation of near-isogenic barley lines. Theor Appl Genet 1993; 86:822–826. doi: 10.1007/BF00212607 24193876 10.1007/BF00212607

[57] Gauch HG, Zobel RW. AMMI analysis of yield trials In: Kang MS, Gauch HG Jr (eds) Genotype-by environment interaction. CRC Press, Boca Raton, pp 85–122, 1996.

[58] Fox PN, Rosielle AA. Reducing the influence of environmental main-effects on pattern analysis of plant breeding environments. Euphytica 1982; 31: 645–656.

[59] Miranda GV, Souza LV, Guimarães M, Lauro JM, Namorato H, Oliveira LR, et al Multivariate analyses of genotype × environment interaction of popcorn. Pesq Agropec Bras 2009; 44(1): 45–50.

[60] Silveira LCI, Kist V, Paula TOM, Barbosa MHP, Peternelli LA, Daros E. AMMI analysis to evaluate the adaptability and phenotypic stability of sugarcane genotypes. Sci Agric. 2013; 70(1): 27–32.

[61] Kleinwächter M, Selmar D. New insights explain that drought stress enhances the quality of spice and medicinal plants: potential applications. Agron. Sustain Dev. 2015; 35(1):121–31.

[62] Akinwale RO, Fakorede MAB, Badu-Apraku B, Oluwaranti A. Assessing the usefulness of GGE biplot as a statistical tool for plant breeders and agronomists. Cereal Res Comm. 2014; 42(3) 534–546.

[63] Gauch HG, Piepho HP, Annicchiarico P. Statistical analysis of yield stability trials by AMMI and GGE: Further consideration. Crop Sci. 2008; 48:866–889.

[64] Sharafzadeh S, Alizadeh O. Some medicinal plants cultivated in Iran. J Appl Pharm Sci. 2012; 2(1): 134–137.

[65] Yan W, Holland JB. A heritability-adjusted GGE biplot for test environment evaluation. Euphytica 2010; 171: 355–369.

[66] Ramakrishna A, Ravishankar GA. Influence of abiotic stress signals on secondary metabolites in plants. Plant Signal Behav. 2011; 6(11): 1720–1731. doi: 10.4161/psb.6.11.17613 22041989 10.4161/psb.6.11.17613 PMC3329344

[67] Roostaei M, Mohammadi R, Amri A. Rank correlation among different statistical models in ranking of winter wheat genotypes. The Crop J. 2014; 2: 154–163.

[68] Wolff K, Van Delden W. Genetic analysis of ecological relevant morphological variability in *Plantago lanceolata* L. I Population characteristics. Heredity 1987; 58: 183–192

